# Inequalities in greenspace access and their associations with all-cause and cause-specific mortality attributable to PM_2.5_: a global analysis from 2000 to 2020

**DOI:** 10.1186/s12889-026-28654-w

**Published:** 2026-07-25

**Authors:** Hong Zhang, Haitao Wang, Wei Ma

**Affiliations:** 1 Department of Academic Research, Cheeloo College of Medicine, Qilu Hospital, Shandong University, Jinan, China; 2 Department of Epidemiology, School of Public Health, Cheeloo College of Medicine, Shandong University, Jinan, 250012 China; 3 Shandong University Climate Change and Health Center, Jinan, China; 4 Shandong Provincial Key Laboratory of Intelligent Monitoring, Early Warning, Prevention and Control for Infectious Diseases, Jinan, China

**Keywords:** Greenspace, PM_2.5_, Mortality, Inequality

## Abstract

**Background:**

As mortality from ambient PM_2.5_ pollution rises and the role of greenspaces in sustainable development grows, understanding relevant national trends and their associations becomes critical. This study aimed to describe the spatiotemporal trends in greenspace access and age-standardized mortality rates (ASMR) attributable to ambient PM_2.5_, and to explore their association.

**Methods:**

Data on ASMR attributable to ambient PM_2.5_ from 2000 to 2020 across 204 countries and territories were obtained from Global Burden of Disease Study (GBD) 2021. ‌Greenspace access metrics were derived from the Global Land Analysis and Discovery dataset and linked to the GBD database through standardized country/territory names and temporal alignment. The estimated annual percentage change (EAPC) was calculated to analyze secular trends in the two data sources and the Gini index was used to assess inequalities in greenspace access.

**Results:**

Globally, all-cause ASMR per 100,000 inhabitants attributable to ambient PM_2.5_ decreased from 63.5 in 2000 to 55.4 in 2020 and greenspace access levels decreased from 37.0% in 2000 to 28.6% in 2020. ASMR attributable to ambient PM_2.5_ was higher in children under 5 years and 60-years-and-older age groups, and North Africa and Middle East were the high-risk areas. Greenspace access levels decreased in all GBD regions except for Oceania (EAPC = 0.18, 95% CI: 0.16 to 0.20). Inequality in greenspace increased by nearly one-fifth in 2020 (Gini: 0.24) compared to 2000 (Gini: 0.20) in countries with a low sociodemographic index. A negative association was observed between greenspace access and PM_2.5_-related mortality for ischemic heart disease, stroke, chronic obstructive pulmonary disease, and neonatal disorders.

**Conclusions:**

Our findings highlight the importance of prioritizing equity-focused green policies to reduce mortality risk associated with ambient PM_2.5_.

**Supplementary Information:**

The online version contains supplementary material available at https://doi.org/10.1186/s12889-026-28654-w.

## Background

Greenspaces, as integral components of ecosystems, provide essential ecosystem services such as environmental purification, microclimate regulation, and the reduction of noise and air pollution [1]. Previous studies have highlighted that exposure to greenspace is associated with reduced all-cause mortality, as well as the morbidity and mortality of a wide range of infectious diseases and cardiovascular diseases [2–4]. In addition, green spaces can increase physical activity, reduce stress, depression, and anxiety, and improve mental health [5]. Meanwhile, inequalities in greenspace exposure and accessibility are receiving increasing attention due to their potential adverse effects on mental and physical health [6, 7]. Although existing studies have identified global inequities in greenspace access [8], the long-term trends of such inequality across countries remain understudied.

Ambient fine particulate matter (PM_2.5_) poses a considerable health risk worldwide. In 2021, approximately 4.7 million premature deaths were attributed to ambient particulate matter pollution [9]. The World Health Organization recommends an annual mean PM_2.5_ concentration of less than 5 µg/m³, however, more than 90% of the world’s population lives in areas that exceed this threshold [10]. PM_2.5_ particles could transport various hazardous substances such as heavy metals and toxins [11]. Exposure to PM_2.5_ has been associated with multiple adverse health outcomes, including declined cardiopulmonary and cognitive function [12, 13], adverse perinatal outcomes [14], and increased mortality risk [15]. To date, the epidemiological dynamics of the mortality burden attributable to PM_2.5_ at global and national levels remain unknown, despite the ongoing increase in global exposure to ambient PM_2.5_. Understanding the spatiotemporal patterns of mortality burden attributable to PM_2.5_ is crucial for developing tailored strategies for global public health actions and risk communication.

Although several studies have examined the relationship between greenspace, mortality and ambient particulate matter pollution [16, 17], few have focused on the association between inequalities in greenspace exposure or access and PM_2.5_-associated cause specific mortality from a global perspective. To fill this evidence gap, we conducted a global-scale analysis to characterize the epidemiological characteristics and temporal trend of greenspace access and the mortality burden attributable to ambient PM_2.5_ over the first two decades of this century. Additionally, we explored the inequalities in greenspace access, and the associations between greenspace access and PM_2.5_ related mortality.

## Methods

### Study design and data sources

We conducted a secondary analysis of the Global Burden of Disease Study (GBD) 2021. Annual data on all-cause and cause-specific age-standardized mortality rate (ASMR) attributable to ambient PM_2.5_ at country level for 204 countries and territories from 2000 to 2020 were downloaded from the online Global Health Data Exchange (http://ghdx.healthdata.org/gbd-results-tool). Details of the GBD 2021 and the specific methods for estimating mortality burden attributable to ambient PM_2.5_ have previously been published [8]. Data on population demographics, socioeconomic conditions, and health services were downloaded from Our World in Data (https://ourworldindata.org/) and United Nations Statistics (http://data.un.org/) to serve as covariates in our models.

We leveraged the global land cover product at 30-m spatial resolution for 2000, 2005, 2010, 2015 and 2020 from The Global Land Analysis and Discovery (GLAD) Landsat Analysis Ready Data (https://glad.umd.edu/dataset). The GLAD dataset includes 13 different land cover general classes with overall accuracy of above 85% for all land cover and land use themes, except for built-up lands. We extracted all types of forest and vegetation cover from the GLAD maps as greenspaces. We filled in the missing greenspace data by spline interpolation. WorldPop (https://www.worldpop.org/) population dataset with 100 m spatial resolution was used to quantify the spatially distribution of population from 2000 to 2020. Various databases were linked based on the standardized names of countries or territories and years.

In this study, number of deaths and age standardized all-cause mortality (per 100 000 population) attributable to ambient PM_2.5_ was the primary outcome, with seven types of cause-specific (ischemic heart disease, stroke, upper respiratory infections, chronic obstructive pulmonary disease, diabetes mellitus type 2, tracheal, bronchus, and lung cancer, or neonatal disorders) mortality attributable to ambient PM_2.5_ as secondary outcomes.

### Statistical analysis

Data on deaths, mortality rate attributable to ambient PM_2.5_ were reported as numbers with 95% uncertainty intervals. Estimated annual percentage changes (EAPC) in deaths, mortality rate due to ambient PM_2.5_ and greenspace access (GA) from 2000 to 2020 were calculated to quantify the secular trends. The following linear regression model was used: y = α + *β*x + ε, where y was the logarithmic indicators and x was the calendar year. EAPC was calculated as (exp (*β*) -1) × 100, and the 95% confidence interval (CI) of EAPC was also calculated using this equation by replacing *β* with the 95% CI of *β*. An increasing trend was considered when the 95% CI of the corresponding EAPC estimation was > 0, decreasing trends if the 95% CI was < 0, and a constant trend was considered if the 95% CI included 0. We calculated EAPCs and their 95% CI from 2000 to 2020 at the country levels.

To ensure spatial consistency between greenspace data and population data, we aggregated the 30 m greenspace binary map into 100 m grids and calculated mean fractional greenspace coverage. A population-weighted exposure model was applied to quantify levels of greenspace access. The population-weighted model considers the density and distribution of population and green spaces, assigning higher weights to greenspace access where more people reside. In short, this approach combines greenspace availability, spatial reachability (defined by buffer distances, a fixed spatial threshold extending outward from each target grid, with values of 100, 500, and 1000 m), and human distribution to reflect human access to greenspace. It provides a useful alternative measure for potential green space access in large-scale epidemiological research. Details of the modeling process are provided in the supplementary methods (Supplementary file 1). The Gini index metric, which ranges from 0 (absolute equality) to 1 (absolute inequality), was employed to assess the inequality in greenspace access.

A LOESS regression on all data from 2000 to 2020 was conducted to define the expected relationship between greenspace access and mortality attributable to ambient PM_2.5_. Additionally, the association between GA and mortality rates (all cause and seven cause specific) was further assessed using the generalized estimating equation model, which used a gamma distribution and loglink function to control for the skewed nature of mortality. The dependent variable is the natural logarithm of the age-standardized mortality rate. We investigated the relationship between GA and mortality rates using crude incidence risk ratios and corresponding 95% confidence intervals. Adjustments were made for year, population density, urbanization rate, average years of schooling, gross domestic product per capita, and human development index. To assess the robustness of the findings, we replaced the human development index with the socio-economic index (SDI, a GBD-derived index stratifying regions by income, education and fertility) in sensitivity analyses. R software (version 4.0.4) was used for the statistical analyses, with the ineq package (version 0.2.13) and geepack package (version 1.3.12) employed for inequality calculation and generalized estimating equation modeling, respectively. A *P* value < 0.05 was regarded as significant.

## Results

### Distribution and temporal trend of mortality attributable to ambient PM_2.5_

**Fig. 1:**
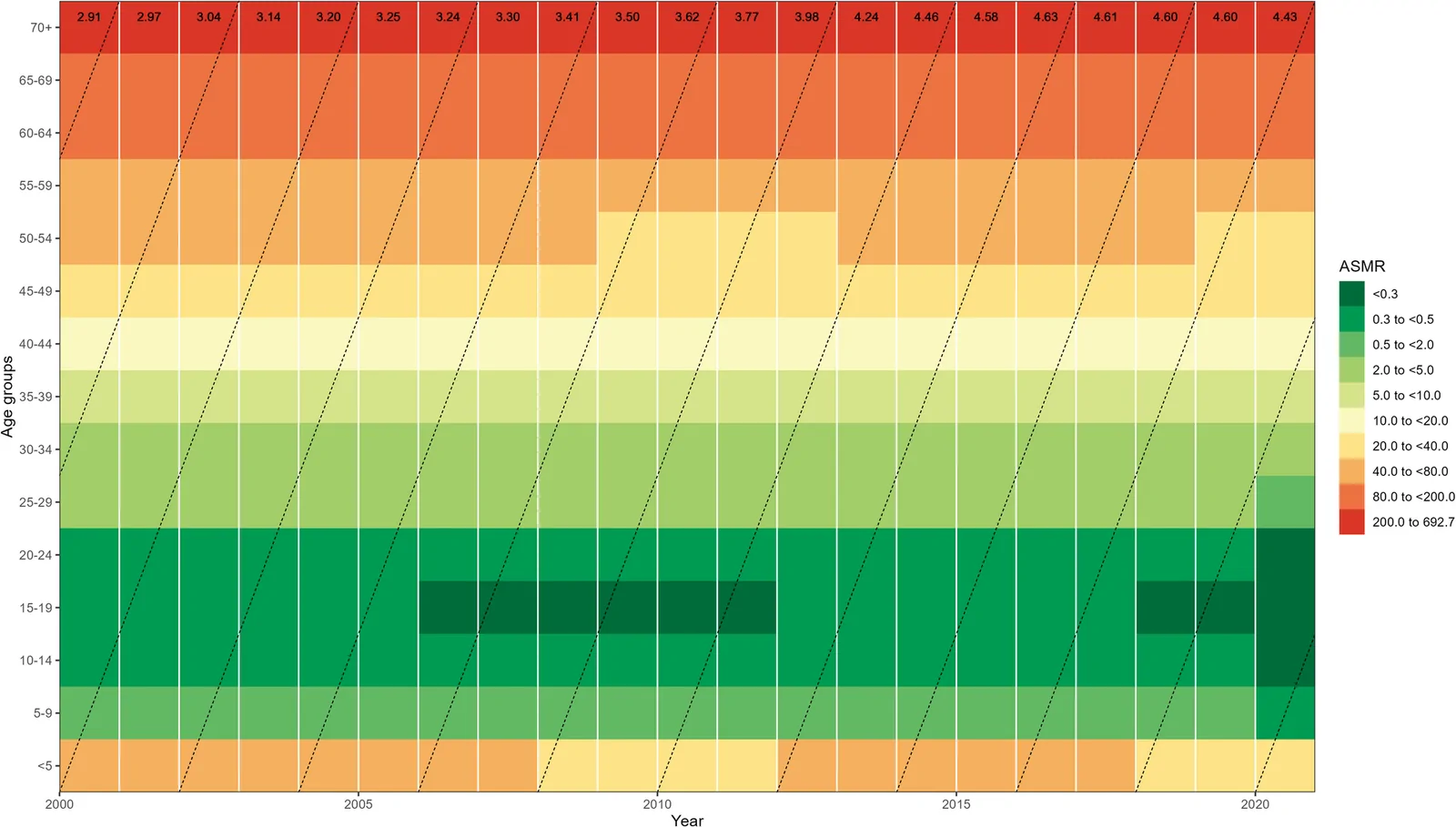
Distribution of mortality rate attributable to ambient PM_2.5_ by age and year, globally. ASMR: age standardized mortality rate. Numbers shown at the top of the figure represent PM2.5-attributed deaths

**Fig. 2:**
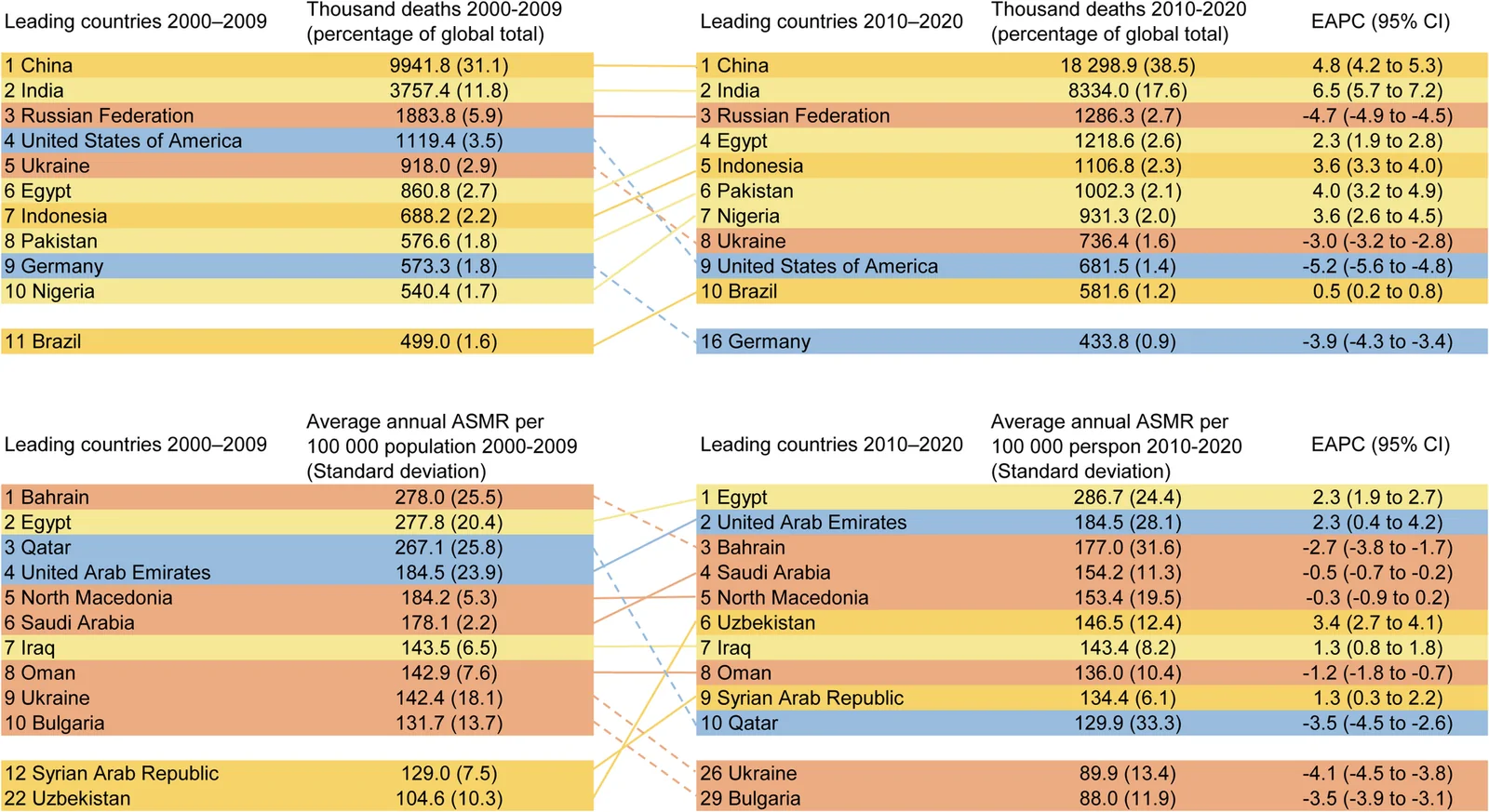
Leading countries with highest all-cause death and mortality attributable to ambient PM_2.5_. ASMR: age standardized mortality rate; EAPC: estimated annual percentage change

Globally, the all-cause ASMR per 10,000 people related to ambient PM2.5 exposure fell from 63.5 in 2000 to 55.4 in 2020, while the number of all-cause deaths attributable to ambient PM_2.5_ increased by 52.2%, from 2.91 million in 2000 to 4.43 million in 2020 (EAPC = 2.7, 95% CI: 2.4 to 3.0) (Supplementary file 2). The highest age-specific all-cause mortality rate attributable to ambient PM_2.5_ occurred in those over 60 years of age group (Fig. 1). All-cause deaths attributable to ambient PM_2.5_ in all leading countries (top ten) present a notable increasing trend during the period 2000–2020, except for Russia, the USA, Ukraine and Germany (Fig. 2). India and China showed a rapid increase in deaths attributable to ambient PM_2.5_, with an EAPC of 6.5 (95% CI: 5.7 to 7.2), 4.8 (95% CI: 4.2 to 5.3), respectively. All top ten countries in all-cause ASMR attributable to ambient PM_2.5_ in 2000–2009 and 2010–2020 were Middle East countries, with four exceptions (North Macedonia, Ukraine, Bulgaria and Uzbekistan). In 2010–2020, the top three countries in annual average ASMR due to ambient PM_2.5_ were Egypt (EAPC = 2.3, 95% CI: 1.9 to 2.7), United Arab Emirates (EAPC = 2.3, 95% CI: 0.4 to 4.2), and Bahrain (EAPC=-2.7, 95% CI: -3.8 to -1.7). The rankings of these attributable mortality metrics changed over time. A notable change was Uzbekistan, which ranked twenty-second in average annual ASMR attributable to ambient PM_2.5_ during 2000–2009, but rose to sixth in 2010–2020 (EAPC = 3.4, 95% CI: 2.7 to 4.1).

**Fig. 3:**
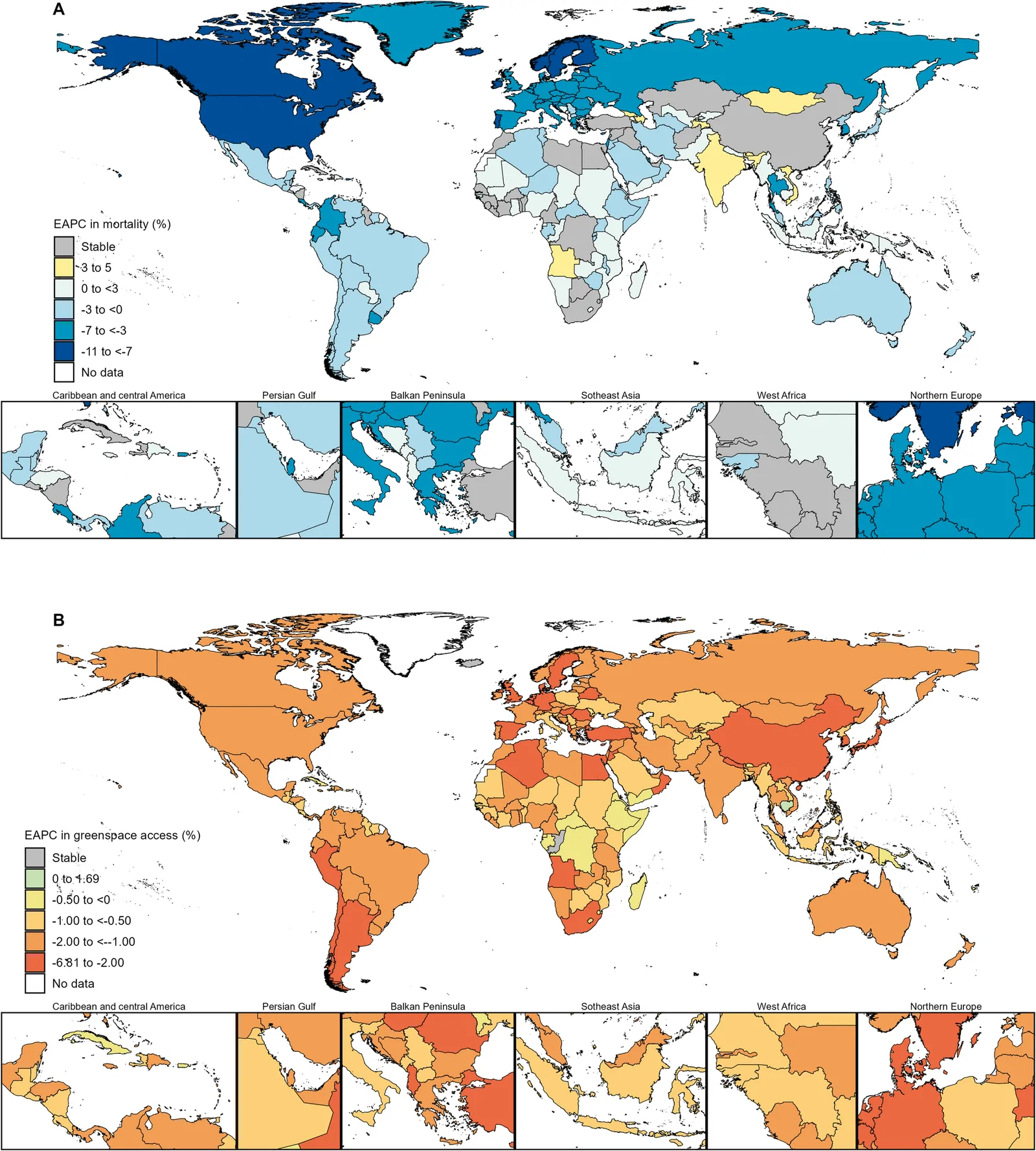
Estimated annual percentage change in all-cause mortality attributable to ambient PM_2.5_ and greenspace access. ASMR: age standardized mortality rate

After controlling the effects of population growth and ageing by converting counts to age-standardized rates, the global all-cause ASMR attributable to ambient PM_2.5_ from 2000 (79.1 per 100 000 population) to 2020 (68.6 per 100 000 population) showed a slight decrease, with an EAPC of -0.3 (95% CI: -0.5 to -0.2) (Table 1). At the GBD regional level, the highest ASMR attributable to ambient PM_2.5_ occurred in Eastern Europe (136.8 per 100 000 population) in 2000 but in North Africa and Middle East (99.5 per 100 000 population) in 2020. The ASMR due to ambient PM_2.5_ increased in Central Asia, South Asia, Southeast Asia, East Asia, and Oceania from 2000 to 2020. South Asia had the fastest increase in ASMR, with an EAPC of 2.4 (95% CI: 2.0 to 2.8). High-income North America had the largest decline in ASMR, with an EAPC of -5.8 (95% CI: -6.2 to -5.3). At the country or territory level, Egypt, Iraq and Bahrain exhibited the highest ASMR attributable to ambient PM_2.5_ in 2020 (Supplementary file 3). Vietnam, Mongolia and India showed rapid increase in ASMR attributable to ambient PM_2.5_, with an EAPC of 5.4 (95% CI: 5.0 to 5.9), 4.8 (95% CI: 3.9 to 5.8), and 3.9 (95% CI: 3.1 to 4.7), respectively (Fig. 3 and Supplementary file 4). The Nordic countries, including Estonia (EAPC=-10.5, 95% CI: -10.9 to -10.1), Finland (EAPC=-9.1, 95% CI: -9.7 to -8.4), and Sweden (EAPC=-8.5, 95% CI: -8.9 to -8.0), experienced the fastest decline in ASMR attributable to ambient PM_2.5_ from 2000 to 2020.

**Table 1:** Age standardized all-cause mortality due to ambient PM_2.5_ and levels of greenspace access by GBD region, for 2000 and 2020, and their percentage change between 2000 and 2020

GBD regions	Mortality rate attributable to PM_2.5_ (per 100 000)			Greenspace access (%)		
	2000	2020	EAPC (95% CI)	2000	2020	EAPC (95% CI)
Global	63.5 (47.7 to 79.1)	55.4 (40.9 to 68.6)	-0.3 (-0.5 to -0.2)	37.0	28.6	-1.33 (-1.38 to -1.28)
Central Asia	76.7 (34.8 to 131.7)	94.3 (66.7 to 124.1)	0.8 (0.3 to 1.2)	42.9	32.3	-1.1 (-1.2 to -1.0)
Central Europe	90.8 (59.0 to 111.2)	47.0 (36.7 to 57.6)	-2.9 (-3.2 to -2.7)	37.1	28.0	-1.3 (-1.5 to -1.2)
Eastern Europe	136.8 (93.5 to 185.7)	44.2 (26.2 to 68.0)	-4.2 (-4.6 to -3.8)	34.1	27.7	-1.06 (-1.11 to -1.02)
High income						
Australasia	10.5 (1.2 to 22.4)	6.3 (3.4 to 9.5)	-2.8 (-3.3 to -2.3)	15.5	11.4	-1.5 (-1.7 to -1.3)
High-income Asia Pacific	22.7 (11.3 to 37.1)	12.7 (7.1 to 19.5)	-3.1 (-3.3 to -2.8)	22.1	11.7	-4.2 (-4.7 to -3.7)
High-income North America	32.1 (24.1 to 41.0)	9.0 (4.9 to 13.6)	-5.8 (-6.2 to -5.3)	17.9	13.8	-1.2 (-1.3 to -1.1)
Southern Latin America	46.1 (30.6 to 60.3)	28.4 (16.4 to 42.0)	-2.4 (-2.6 to -2.2)	30.8	21.3	-2.3 (-2.5 to -2.0)
Western Europe	39.5 (31.4 to 47.6)	11.6 (8.0 to 15.8)	-5.3 (-5.5 to -5.1)	19.0	13.1	-2.1 (-2.2 to -1.9)
Latin America and Caribbean						
Andean Latin America	65.3 (39.1 to 93.7)	36.8 (22.7 to 53.2)	-2.7 (-2.9 to -2.4)	59.1	40.6	-2.0 (-2.1 to -1.9)
Caribbean	43.8 (18.8 to 74.2)	41.1 (21.5 to 62.5)	-0.6 (-0.8 to -0.4)	50.9	44.2	-0.7 (-0.8 to -0.6)
Central Latin America	57.3 (42.1 to 72.1)	32.8 (23.8 to 42.4)	-2.6 (-2.7 to -2.4)	47.0	36.7	-1.3 (-1.4 to -1.2)
Tropical Latin America	38.9 (20.1 to 65.3)	22.5 (12.8 to 34.0)	-2.4 (-2.5 to -2.2)	32.3	23.3	-1.8 (-1.9 to -1.7)
North Africa and Middle East	116.0 (93.2 to 138.0)	99.5 (79.8 to 118.6)	-0.3 (-0.4 to -0.2)	34.3	23.5	-1.9 (-2.1 to -1.7)
South Asia	50.4 (34.0 to 71.3)	76.7 (48.4 to 103.2)	2.4 (2.0 to 2.8)	32.6	23.4	-1.6 (-1.7 to -1.5)
East Asia	92.2 (52.2 to 142.7)	96.0 (66.3 to 118.5)	1.5 (1.1 to 1.9)	38.5	25.3	-2.3 (-2.4 to -2.1)
Oceania	35.4 (11.4 to 83.4)	37.0 (11.6 to 88.5)	0.4 (0.3 to 0.6)	78.0	80.5	0.18 (0.16 to 0.20)
Southeast Asia	51.8 (28.1 to 79.7)	57.9 (37.0 to 78.5)	0.8 (0.6 to 0.9)	41.9	34.6	-0.96 (-1.04 to -0.87)
Sub-Saharan Africa						
Central sub-Saharan Africa	28.8 (17.3 to 45.1)	35.5 (20.3 to 53.0)	0.5 (0.2 to 0.8)	83.4	67.4	-1.00 (-1.03 to -0.97)
Eastern sub-Saharan Africa	19.5 (12.2 to 28.3)	18.7 (11.7 to 27.8)	0.0 (-0.1 to 0.2)	62.7	54.2	-0.73 (-0.76 to -0.71)
Southern sub-Saharan Africa	65.9 (42.6 to 93.4)	63.1 (43.7 to 82.4)	0.9 (0.5 to 1.2)	48.7	32.9	-1.9 (-2.0 to -1.8)
Western sub-Saharan Africa	53.9 (31.9 to 83.6)	57.0 (30.8 to 85.8)	0.5 (0.1 to 0.9)	60.7	48.7	-1.07 (-1.09 to -1.04)

### Distribution and temporal trend of greenspace access

Globally, the values of GA showed a slight decrease during the period 2000 (37.0%) to 2020 (28.6%), with an EAPC of -1.33 (95% CI: -1.38 to -1.28) (Table 1). At the GBD regional level, greenspace access levels decreased in all regions, except for Oceania (EAPC = 0.18, 95% CI: 0.16 to 0.20). The highest level of greenspace access occurred in Central sub-Saharan Africa in 2000 (83.4%) and in Oceania in 2020 (67.9%), while Australasia had the lowest level of greenspace access (15.5% in 2000 and 11.4% in 2020). At the country level, the top three countries for GA levels in 2020 were South Sudan (97.9%), Papua New Guinea (92.9%), and Equatorial Guinea (88.6%), all situated near the equator (Supplementary file 3). In 2020, the GA values for the top 10 populated countries are 25.2% (China), 23.9% (India), 14.2% (US), 39.8% (Indonesia), 20.1% (Pakistan), 22.7% (Brazil), 42.2% (Nigeria), 21.7% (Bangladesh), 18.8% (Russia), and 28.9% (Mexico). In 2000–2020, Bahrain, Singapore, and Japan had the fastest decline in GA, with an EAPC of -6.8 (95% CI: -7.4 to -6.3), -5.5 (95% CI: -6.7 to -4.4) and − 4.7 (95% CI: -5.4 to -4.1), respectively (Fig. 3 and Supplementary file 5). Maldives and Cambodia had the fastest increase in GA, with an EAPC of 1.7 (95% CI: 0.8 to 2.6) and 1.0 (95% CI: 0.9 to 1.1), respectively.

### Socioeconomic disparities in greenspace access

**Fig. 4:**
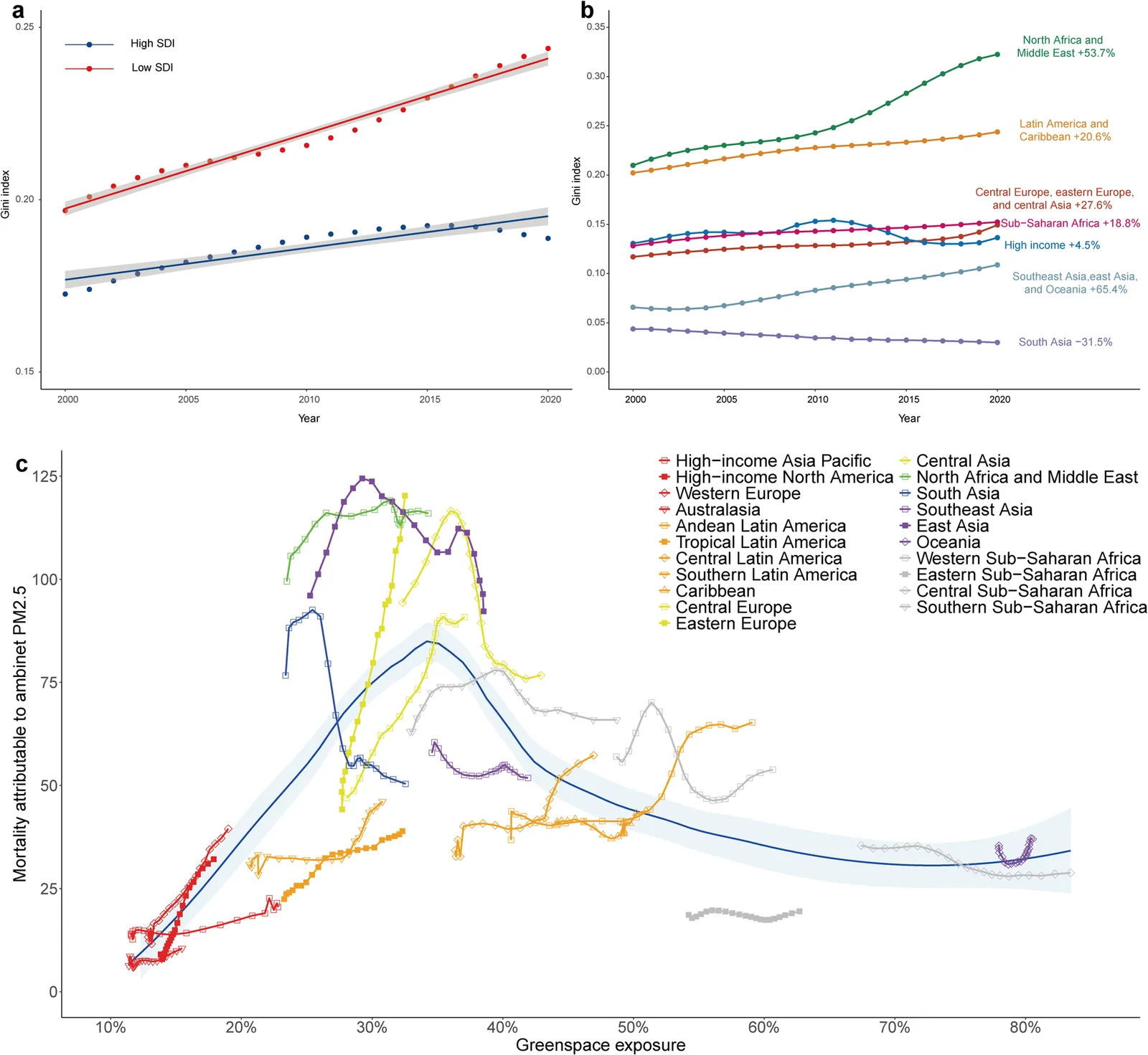
Interannual variation in greenspace access inequality and its association with mortality due to ambient PM_2.5_. Gini coefficients of greenspace access across different SDI regions (**a**) and GBD super-regions (**b**). The blue line represents the expected value of mortality rate based on a LOESS regression of all years of estimates by GBD locations and their greenspace access value (**c**)

The interannual variation in the Gini coefficients of greenspace access across different SDI regions and GBD regions are shown in Fig. 4. Globally, a significant increase in greenspace access inequality was observed over time in low SDI regions (*P* < 0.001 for trend, EAPC = 1.0, 95% CI: 0.9 to 1.1), paired with an increase in the Gini coefficients from 0.20 in 2000 to 0.24 in 2020 (Supplementary file 6). The Gini coefficients of greenspace access in high SDI regions showed a slight increase (*P* < 0.001 for trend, EAPC = 0.5, 95% CI: 0.4 to 0.6). At the GBD region level, inequality of greenspace access was highest in North Africa and Middle East in 2020, and a significant increase was observed from 2000 to 2020 (*P* < 0.001 for trend, EAPC = 2.1, 95% CI: 1.9 to 2.4). Inequalities in greenspace access increased rapidly in Southeast Asia, east Asia, and Oceania, with a 65.4% increase in 2020 compared with 2000 (*P* < 0.001 for trend, EAPC = 2.9, 95% CI: 2.7 to 3.1).

### Effect of greenspace access on PM_2.5_-related mortality

**Fig. 5:**
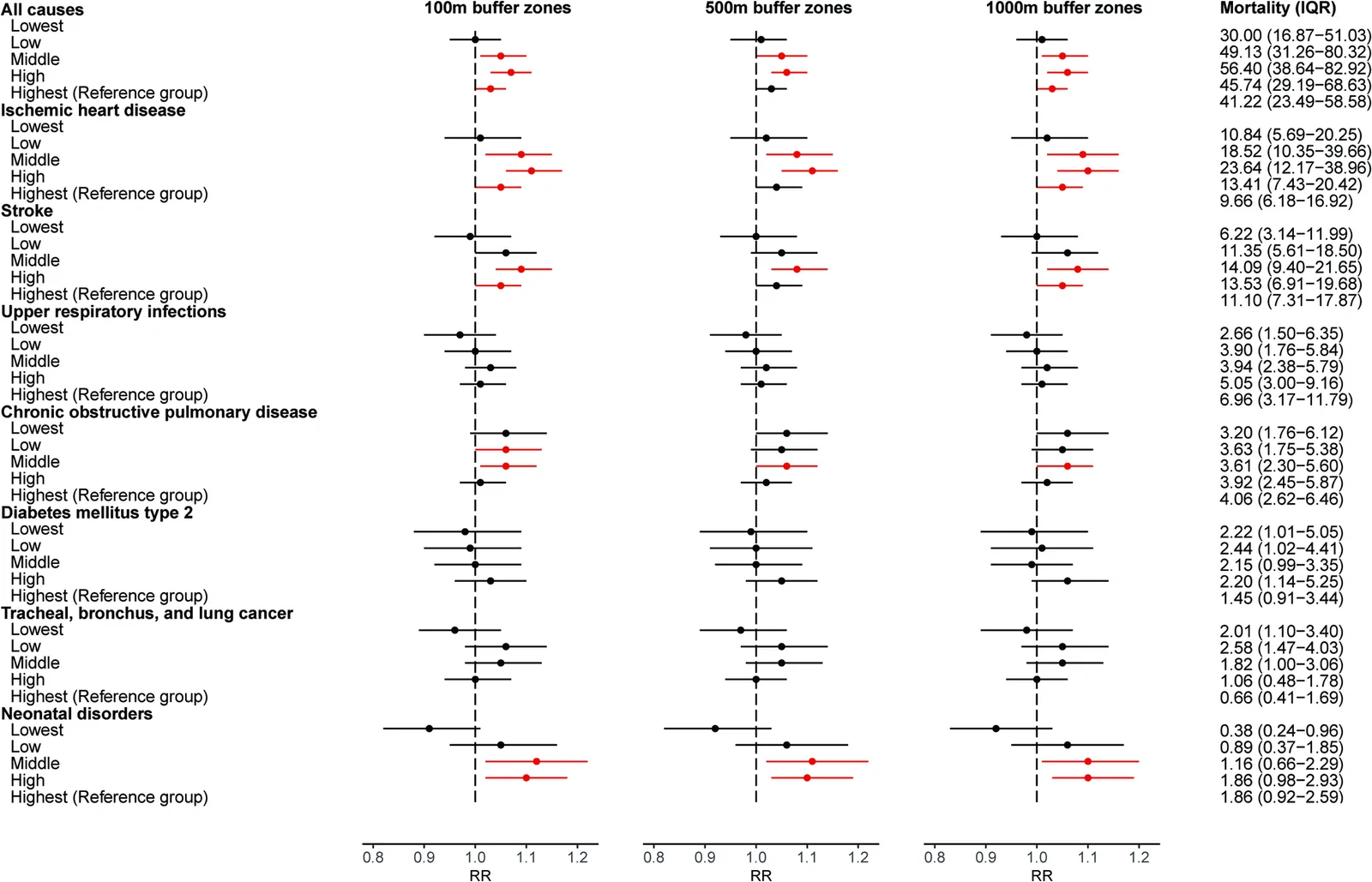
Association between greenspace access and all cause and cause-specific mortality rate attributable to ambient PM_2.5_

Figure 4c shows that all-cause age-standardized mortality attributable to ambient PM_2.5_ demonstrates a bell-shaped relationship with human access to greenspace. In the multivariable generalized estimating equation model, a negative association was observed between GA levels and all-cause mortality, with the highest GA levels as reference group (low: adjusted mortality risk ratio 1.05, 95% CI: 1.00 to 1.10; middle: 1.06, 95% CI: 1.03 to 1.10) (Fig. 5). Negative associations were also found between GA levels and particular types of cause-specific mortality, including ischemic heart disease, stroke, chronic obstructive pulmonary disease, and neonatal disorders. The mortality risk due to ambient PM_2.5_ of ischemic heart disease (low: 1.08, 95% CI: 1.02 to 1.15; middle: 1.11, 95% CI: 1.05 to 1.17), stroke (middle: 1.09, 95% CI: 1.03 to 1.14), chronic obstructive pulmonary disease (middle: 1.06, 95% CI: 1.00 to 1.12), and neonatal disorders (middle: 1.11, 95% CI: 1.02 to 1.22; high: 1.11, 95% CI: 1.03 to 1.19) was much higher in areas with high, middle, or low GA levels than in areas with the highest GA levels. Sensitivity analysis showed that the associations between greenspace access and mortality were similar (Supplementary file 7).

## Discussion

Integrating data from the Global Burden of Disease database and global Land cover dataset, we qualified the association between greenspace access and all cause and cause specific mortality globally from 2000 to 2020. The total number of deaths attributable to ambient PM_2.5_ increases by over 50% in 2020 compared to 2000, but the corresponding age-standardized mortality rates decreases slightly. We observed inequalities in greenspace accessibility, with these disparities being more pronounced and increasing at a faster rate in countries and territories ranking lower on the sociodemographic index. Inequalities in greenspace access have increased over time, particularly pronounced in North Africa and Middle East. We also found that individuals exposed to lower greenspace supply were at increased risk of mortality from all causes and from the four cause-specific outcomes (ischemic heart disease, stroke, chronic obstructive pulmonary disease, and neonatal disorders). The study findings provide evidence for prioritizing greening policies to mitigate the potential risk of mortality caused by atmospheric particulate pollution.

Ambient PM_2.5_ air pollution is a main contributor of mortality in the population. In the past two decades, the estimated global deaths attributable to ambient PM_2.5_ has risen by over 50%, despite a modest decline in age-standardized mortality rates caused by ambient PM_2.5_. Recently, several systematic reviews have suggested that short-term and long-term exposure to ambient air pollution was associated with premature mortality [18, 19]. A global time-series analysis in 652 cities indicated that each 10 µg/m³ rise in the 2-day moving average of PM_2.5_ concentration was associated with a 0.68% increase (95% CI: 0.59–0.77) in daily all-cause mortality [20]. Results from seven large European cohorts showed that long-term exposure to concentrations of PM_2.5_ lower than the WHO 2005 air quality guideline values for PM_2.5_ (10 µg/m^3^) was associated with increased risk of non-accidental mortality, with a hazard ratio of 1.078 (95% CI: 1.046 to 1.111) per 5 µg/m^3^ increment in PM_2.5_ [21]. The central biological mechanism of PM_2.5_ exposure leading to cause-specific mortality involves oxidative stress and systemic inflammation, which can ultimately result in cellular damage or apoptosis [22, 23]. Therefore, continuous improvements in air quality are essential in reducing the burden of mortality.

Our study showed that the burden of mortality attributable to ambient PM_2.5_ was heavier among children under 5 years and the elderly population. This observation may be primarily driven by the higher baseline mortality rates in these age groups. Due to the unavailability of age- and sex-specific exposure-response functions for PM_2.5_-attributable mortality, our findings reflect distributional differences in mortality burden across age groups rather than true variations in vulnerability to PM_2.5_ exposure. Young children under 5 years are particularly vulnerable to ambient air pollution due to their small airways, immature respiratory and immune systems, and faster breathing [24]. Quantitative goals to reduce under-five mortality is a key objective of Sustainable Development Goal 3 set by United Nations, especially in developing regions. A study conducted in South Asia estimated that the number of under-5 deaths attributable to PM_2.5_ is projected to increase by 36.6% during 2045–2049 under current air pollution and climate change scenario [25]. Thus, effective implementation of robust climate change mitigation and air pollution control policies is required. On the other hand, previous studies have provided robust and consistent evidence that elderly are susceptible to the harmful effects triggered by particulate matter exposure [26]. PM_2.5_ particles could penetrate the respiratory system and directly enter the bloodstream and specific organs, aggravating local oxidative stress and inflammation, and consequently impair lung function [27]. Another mechanism is considered that PM_2.5_ exposure could induce pathological changes and ultra-structural damage in hearts, including mitochondrial swell and cristae disorder, oxidative stress, and cellular homeostasis imbalance [28]. Older adults have relatively decreased immunologic function and higher prevalence of comorbidities [29], and they may be exposed to pollutants and many other health hazards for longer periods of time [27], thus the adverse effects of air pollution are expected to be more severe in older adults than in younger adults. Meanwhile, previous studies have consistently demonstrated that higher green space exposure is linked to a lower mortality risk among older adults [30]. The world’s population is ageing with those aged over 60 years will reach 2.1 billion by 2050 and two-thirds live in lower and middle income countries [31, 32]. With the global demographic shift towards an ageing population, which is closely associated with increasing health and social care needs, equitable public policies focused on enhancing air quality and promoting green space access to provide environmental support for healthy ageing are needed.

Our study revealed disparities in greenspace distribution across countries with varying sociodemographic index. Globally, the inequality of greenspace access was higher in low SDI region and a significant increase was noted. Consistent with our findings, Chen et al. discovered that greenspace exposure inequality in Global South cities is nearly twice that observed in Global North cities [33]. Green space, as an important public natural resource, is recognized for its positive impacts on both health and social well-being. The growing disparities in their distribution may exacerbate health and social inequalities for residents, especially in countries at a lower level of SDI. At the GBD region level, we also found evidence that the inequality of greenspace access was most pronounced in North Africa and the Middle East. The geoclimatic characteristics in this region are mainly characterized by a high percentage of arid and semiarid lands, and an increased drought risk and an intensification of drought periods due to rising global temperatures [34]. Such climates render this region unsuitable for the growth of green vegetation, consequently escalating the costs associated with greenspace provision and maintenance, as well as heightening the likelihood of unequal distribution of greenspaces [35]. Additionally, urbanization may also influence the inequality of greenspace exposure. Urbanization is a dominant global demographic trend and it is estimated that approximately 75% of the world’s population will reside in urban areas by 2050 [36]. The destruction effects of urbanization on greenspace exposure include the conversion of green land cover to build land cover in the early-stage of urban development [37]. Rapid urban expansion may result in the conversion of vegetated areas into impervious surfaces or buildings, thereby leading to a subsequent reduction in greenspace coverage. On the other hand, the demand and supply of greenspace are likely to increase as the prosperity of citizens increases as urban development progresses, which may include initiatives such as planting street trees and creating vertical gardens [7, 38]. These findings highlight the need for prioritizing greening policies to mitigate green access disparity and achieve sustainable development goals in the context of urbanization.

We found a significant negative association between greenspace access and mortality attributable to PM_2.5_, and the health effect of greenspace access were more pronounced for some types of cause-specific mortality. In line with our findings, a prospective cohort study of 2,185,170 adults aged 30 years and older in Belgium found that an interquartile increase in greenspace exposure (defined using NDVI within buffers of 500 m) was associated with a decreased risk of non-accidental mortality (hazard ratio (HR) 0.97, 95% CI 0.96–0.98) [39]. Data from the UK Biobank cohort study of 232,926 participants (all-cause mortality: HR 0.94, 0.91 to 0.97, non-injury mortality: HR 0.95, 0.92 to 0.97) [3], the ELAPSE pooled cohort comprised 327,388 individuals from six European countries (natural-cause mortality: HR 0.95, 0.94 to 0.96) [40], and a cohort study of 574,830 adults in Canada (non-accidental mortality: HR 0.95, 0.94 to 0.96 ) showed statistically significant benefits of green surroundings for mortality [41]. Findings from a meta-analysis indicated that increased exposure to greenness was correlated with a decreased risk of all-cause mortality and stroke mortality among older adults [42]. In addition, a review article indicated that living in areas with high surrounding greenness during pregnancy was associated with a lower risk of neonatal low birth weight, and higher exposure to greenness during childhood was associated with a decreased risk of obesity and neurodevelopmental issues [43]. A statistically non-significant association between residential greenspace exposure and all-cause mortality was, however, reported in a longitudinal study in London that included 4,645,581 participants aged 18 years and older (HR 1.0004, 0.9996 to 1.0012) [2]. Green space exposure has been inversely associated with cardiometabolic risk factors, including elevated fasting blood glucose, elevated triglycerides, and hypertension [44]. Further mechanisms underlying the association between green space exposure and lower cardiovascular disease risk include diminished exposure to air pollution, noise, and extreme heat, alongside fostered healthier lifestyles, strengthened social cohesion, and boosted psychological resilience [45]. It’s important to note that the health-beneficial effects of greenspaces not only in terms of quantity but also quality. The quality of green space, including park design, nature conservation, facilities and features that offer recreational and ecological benefits, also plays a crucial role in relation to the effects on health [46]. To better understand the role of greenspace on mortality, future research might wish to incorporate the perceived evaluations of residents on greenspaces quality.

Our current study has several advantages. First, to our knowledge, this is the first study to evaluate global spatiotemporal trends and inequalities in greenspace access at a global scale. The findings could help detect vulnerable hotspots of greenspace access worldwide and support local authorities in prioritizing greenspace enhancement and mitigating environmental disparities. Second, the association between greenspace access and all cause and cause specific mortality attributable to ambient PM_2.5_ might contribute toward a more complete understanding of the impact of ambient particulate matter on health consequences. Several limitations should be noted. First, greenspace access estimates at 30-m resolution may not be able to identify certain greenspace types in small and fragmented patches, such as street plantation and pocket parks. Additionally, the buffer distance method adopted to quantify greenspace reachability does not account for real-world barriers to actual pedestrian accessibility, including varying traffic conditions, heterogeneous road network characteristics (e.g., arterial roads and one-way streets), and greenspace usage constraints (e.g., opening hours, entry restrictions on gated green areas), which may lead to the overestimation or underestimation of actual greenspace access. Moreover, the spatial distributions of greenspace and population footprints are static within each year in this study, we did not consider the dynamic spatiotemporal interactions between greenspace and humans. Second, PM_2.5_ is a complex mixture of various organic and inorganic substances with different physicochemical properties and toxicity, and there are significant differences in PM_2.5_ constituents across different levels of socioeconomic regions [47]. Thus, a more precise assessment of the mortality burden attributable to ambient PM_2.5_ is needed. Third, this study relied on country and territory-level socioeconomic data, which constrained the assessment of intra-country or individual-level socioeconomic inequality. Our greenspace access measurement captures potential spatial access rather than individual-level exposure patterns, which might limit the precision of our greenspace-outcome association estimates. Therefore, integrating individual-level greenspace usage data (such as visitation frequency and duration) into the exposure assessment model would help more accurately quantify the health effects of greenspace. Further research incorporating more granular micro-socioeconomic and individual data is needed to fill the knowledge gap within the field.

## Conclusions

The mortality burden attributable to ambient PM_2.5_ has been slightly decreased, while the death number increased during the past two decades. We observe a contrast in the rate of increase in greenspace access inequality between countries and territories, noting a faster rate in low SDI regions. Furthermore, this study also found a negative association between greenspace access and mortality rate attributable to ambient PM_2.5_. Our results will provide support for the government and public health professionals to upgrade the low levels of health facilitating greenspace and the management of air quality.

## Supplementary Information

Supplementary Material 1.

Supplementary Material 2.

Supplementary Material 3.

Supplementary Material 4.

Supplementary Material 5.

Supplementary Material 6.

Supplementary Material 7.

## Data Availability

All data in the study are available at https://ghdx.healthdata.org/gbd-2021 , http://data.un.org/ , https://ourworldindata.org/ , https://glad.umd.edu/dataset/GLCLUC2020.

## Abbreviations

ASMR: Age standardized mortality rate
GBD: Global Burden of Disease
EAPC: Estimated annual percentage change
GA: Greenspace access
CI: Confidence interval
