# Supplementary material for: Inequalities in greenspace access and their associations with all-cause and cause-specific mortality attributable to PM2.5: a global analysis from 2000 to 2020
Source: BMC Public Health. 2026 Jul 25;26:3293. doi: 10.1186/s12889-026-28654-w (PMC13749990; doi:10.1186/s12889-026-28654-w)
Supplement: Supplementary file 1 [file 12889_2026_28654_MOESM1_ESM.docx]

**Additional file 1.** Estimation of mortality attributable to ambient PM_2.5_ and human greenspace access.

1. Data sources

We collected yearly data on all-cause and cause specific mortality from the Global Health Data Exchange (http://ghdx.healthdata.org/gbd-results-tool) including: (1) global age-standardized mortality rate attributable to ambient PM_2.5_, age-specific mortality attributable to ambient PM2.5 (per 100,000 persons) and deaths numbers attributable to ambient PM_2.5_ from 2000 to 2020; (2) Regional age-standardized mortality rate attributable to ambient PM_2.5_ from 2000 to 2020 by GBD regions; (3) National age-standardized mortality rate attributable to ambient PM_2.5_ from 2000 to 2020. Moreover, we also collected the socio-demographic index (SDI) from the GBD 2021.

The demographic characteristics, socioeconomic conditions, and health services data used as covariates for the generalized estimating equation model were downloaded from the United Nations Statistics (http://data.un.org/) and Our World in Data (https://ourworldindata.org/). Variables included in the study include population density (people per km^2^), percentage of the population living in urban areas (%), average years of schooling (average number of years people aged 25+ participated in formal education), GDP per capita (measured in constant 2011 international-$), and human development index (HDI). HDI is a comprehensive indicator that summarizes key dimensions of human development, including a long and healthy life, access to quality education, and a decent standard of living. Higher values on the index indicate higher levels of human development. Global land cover product for 2000, 2005, 2010, 2015 and 2020 is obtained from The Global Land Analysis and Discovery (GLAD) Landsat Analysis Ready Data (https://glad.umd.edu/dataset). We filled in the missing greenspace data by spline interpolation. Population dataset is from WorldPop (https://www.worldpop.org). The global administrative unit layers are from the Food and Agriculture Organization of the United Nations (https://data.apps.fao.org). Various databases were linked based on country/territory names and years.

2. Estimation of mortality burden attributable to ambient PM_2.5_ in GBD 2021

The specific methods for estimating mortality burden attributable to ambient PM_2.5_ in GBD 2021 have been described in detail previously [1]. In brief, ambient PM_2.5_ exposure was estimated using data from multiple sources, including satellite observations of aerosols in the atmosphere, ground monitor measurements, chemical transport model simulations, population estimates, and land-use data. The cause-specific risk curves were created using meta-regression—Bayesian, regularized, trimmed (MR-BRT) meta-regression tool based on literature review. A comparative risk assessment was conducted to calculate the attributable burden of disease from potential risk factors in the GBD study, and a proportional population attributable fraction approach was used to quantify the contribution of ambient PM_2.5_ exposure to cause-specific mortality burden [2]. In GBD 2021, uncertainty is propagated throughout all the modelling processes. Each estimation was used to model 1000 draws of densities for mortality across locations. The lower and upper bounds of the 95% UI for each indicator in the GBD study was estimated based on the 25th and 975th values of the ordered 1000 values.

The SDI is a composite indicator of socio-demographic development status, with higher values indicating greater socioeconomic development. It is the geometric mean of 0 to 1 indices of mean education for individuals aged 15 years or older, lag-distributed income per capita, and total fertility rate among those under 25 years old. Details can be seen on the page 47-48, Appendix 1 of the reference 1.^1^ In this study, we collected the SDI data for 204 available countries and territories from the GBD 2021 for the years 2000 to 2020. We calculated the mean SDI values for each country or territories and categorized them into two groups, high SDI and low SDI, based on the median. We then calculated the Gini coefficients of greenspace exposure for two SDI groups (high SDI and low SDI) and seven GBD super-regions (Central Europe, Eastern Europe, and Central Asia; High-income; Latin America and Caribbean, North Africa and Middle East; South Asia; Southeast Asia, East Asia, and Oceania; Sub-Saharan Africa).

3. Estimation of greenspace access

The population-weighted exposure model was used to assess the spatial interaction between population and greenspace, with higher weights were allocated to greenspace exposure in areas with higher population density.^3^ Greenspace access was calculated as follows:

$${GE}^{d}=\frac{\sum_{i=1}^{N} P_{i}*G_{i}^{d}}{\sum_{i=1}^{N} P_{i}}$$

where *P_i_* is the population of grid *i*, $\text{G}_{\text{i}}^{\text{d}}$ represents the fractional greenspace coverage of grid i considering nearby green environments with a buffered distances of d (i.e., 100, 500, and 1000 m), N refers to the total number of grids within the specific administrative unit, and GE denotes the population-weighted human greenspace access.

Gini index was utilized to measure the global and regional inequality in human greenspace access, and the derivation process of Gini index have previously been published [3,4]. The Gini coefficient ranges from 0 (absolute equality) to 1 (absolute inequality), with higher values indicating greater inequality in greenspace access. The Gini index can be formulated as:

$$Gini=1-\frac{2*\sum_{i=1}^{n} \sum_{i=1}^{i} g_{i}}{n*\sum_{i=1}^{n} g_{i}}$$

where n is the total population within a country or territory during a pre-set timeframe; g_i_ is the average level of greenspace access of individual *i*.

eReferences

1.	Global burden and strength of evidence for 88 risk factors in 204 countries and 811 subnational locations, 1990-2021: a systematic analysis for the Global Burden of Disease Study 2021. *Lancet*. 2024,403(10440):2162-2203. doi:10.1016/S0140-6736(24)00933-4

2.	Yang X, Zhang T, Zhang Y, et al. Global burden of COPD attributable to ambient PM_2.5_ in 204 countries and territories, 1990 to 2019: A systematic analysis for the Global Burden of Disease Study 2019. *Sci Total Environ*. 2021,796:148819. doi:10.1016/j.scitotenv.2021.148819

3.	Chen B, Wu S, Song Y, et al. Contrasting inequality in human exposure to greenspace between cities of Global North and Global South. *Nat Commun.* 2022,13(1):4636. doi:10.1038/s41467-022-32258-4

4.	Song Y, Chen B, Ho H C, et al. Observed inequality in urban greenspace exposure in China. *Environ Int*. 2021,156:106778. doi:10.1016/j.envint.2021.106778
