# Supplementary material for: Inequalities in greenspace access and their associations with all-cause and cause-specific mortality attributable to PM2.5: a global analysis from 2000 to 2020
Source: BMC Public Health. 2026 Jul 25;26:3293. doi: 10.1186/s12889-026-28654-w (PMC13749990; doi:10.1186/s12889-026-28654-w)
Supplement: Supplementary file 2 [file 12889_2026_28654_MOESM2_ESM.docx]

**Additional file 2*.*** Global deaths and age-standardized mortality rate attributable to ambient PM_2.5_, 2000-2020.

Note: ASMR, age-standardized mortality rate. Shaded sections indicate 95% uncertainty intervals**.**
