# Supplementary material for: Inequalities in greenspace access and their associations with all-cause and cause-specific mortality attributable to PM2.5: a global analysis from 2000 to 2020
Source: BMC Public Health. 2026 Jul 25;26:3293. doi: 10.1186/s12889-026-28654-w (PMC13749990; doi:10.1186/s12889-026-28654-w)
Supplement: Supplementary file 4 [file 12889_2026_28654_MOESM4_ESM.docx]

**Additional file 4.** Estimated annual percentage change (EAPC) in all-cause mortality attributable to ambient PM_2.5_, 2000-2020.

| **GBD regions** | **Countries and territories** | **Mortality attributable to ambient PM_2.5_ (per 100 000 population)** | | **EAPC (%, 95% CI)** | ***P* value*** |
| --- | --- | --- | --- | --- | --- |
|  |  | **2000** | **2020** |  |  |
| Central Asia | Armenia | 77.12 | 92.02 | 1.43 (0.51, 2.36) | 0.010 |
| Central Asia | Azerbaijan | 57.93 | 100.94 | 4.10 (2.75, 5.47) | <0.001 |
| Central Asia | Georgia | 36.30 | 52.62 | 3.27 (2.36, 4.19) | <0.001 |
| Central Asia | Kazakhstan | 100.80 | 89.52 | -0.16 (-0.57, 0.25) | 0.499 |
| Central Asia | Kyrgyzstan | 47.37 | 54.79 | 2.21 (1.58, 2.85) | <0.001 |
| Central Asia | Mongolia | 47.91 | 91.79 | 4.85 (3.88, 5.82) | <0.001 |
| Central Asia | Tajikistan | 39.58 | 65.16 | 3.73 (2.89, 4.59) | <0.001 |
| Central Asia | Turkmenistan | 122.81 | 103.65 | -0.35 (-0.63, -0.06) | 0.031 |
| Central Asia | Uzbekistan | 94.61 | 125.93 | 2.67 (1.90, 3.45) | <0.001 |
| Central Europe | Albania | 38.38 | 48.42 | 1.45 (0.82, 2.08) | <0.001 |
| Central Europe | Bosnia and Herzegovina | 59.00 | 72.01 | 0.88 (0.42, 1.34) | 0.001 |
| Central Europe | Bulgaria | 147.60 | 69.03 | -3.79 (-3.95, -3.62) | <0.001 |
| Central Europe | Croatia | 83.42 | 39.21 | -4.06 (-4.51, -3.61) | <0.001 |
| Central Europe | Czechia | 77.84 | 32.67 | -4.66 (-5.20, -4.12) | <0.001 |
| Central Europe | Hungary | 83.47 | 39.53 | -3.69 (-4.23, -3.15) | <0.001 |
| Central Europe | Montenegro | 80.28 | 82.72 | 0.35 (0.01, 0.70) | 0.057 |
| Central Europe | North Macedonia | 189.33 | 120.47 | -1.92 (-2.37, -1.47) | <0.001 |
| Central Europe | Poland | 85.61 | 41.15 | -3.65 (-4.12, -3.17) | <0.001 |
| Central Europe | Romania | 90.02 | 45.30 | -3.71 (-4.15, -3.27) | <0.001 |
| Central Europe | Serbia | 132.23 | 82.22 | -2.43 (-2.86, -2.00) | <0.001 |
| Central Europe | Slovakia | 90.55 | 44.75 | -3.61 (-4.11, -3.10) | <0.001 |
| Central Europe | Slovenia | 49.61 | 17.65 | -5.36 (-5.66, -5.07) | <0.001 |
| Eastern Europe | Belarus | 150.90 | 63.83 | -4.30 (-4.59, -4.02) | <0.001 |
| Eastern Europe | Estonia | 50.36 | 5.35 | -10.51 (-10.95, -10.06) | <0.001 |
| Eastern Europe | Latvia | 86.19 | 30.53 | -5.18 (-5.73, -4.62) | <0.001 |
| Eastern Europe | Lithuania | 73.62 | 22.11 | -5.33 (-5.90, -4.75) | <0.001 |
| Eastern Europe | Republic of Moldova | 42.00 | 36.79 | 0.11 (-0.71, 0.94) | 0.725 |
| Eastern Europe | Russian Federation | 130.78 | 36.70 | -6.21 (-6.45, -5.97) | <0.001 |
| Eastern Europe | Ukraine | 169.35 | 67.67 | -4.30 (-4.44, -4.17) | <0.001 |
| Australasia | Australia | 10.54 | 6.57 | -1.52 (-2.40, -0.63) | 0.003 |
| Australasia | New Zealand | 10.41 | 4.90 | -2.57 (-3.20, -1.93) | <0.001 |
| High-income Asia Pacific | Brunei Darussalam | 18.43 | 14.60 | 0.31 (-0.98, 1.61) | 0.570 |
| High-income Asia Pacific | Japan | 17.05 | 9.82 | -2.25 (-2.76, -1.74) | <0.001 |
| High-income Asia Pacific | Republic of Korea | 61.68 | 27.69 | -3.81 (-4.32, -3.30) | <0.001 |
| High-income Asia Pacific | Singapore | 51.46 | 15.81 | -4.53 (-5.23, -3.84) | <0.001 |
| High-income North America | Canada | 19.19 | 4.91 | -7.56 (-7.96, -7.17) | <0.001 |
| High-income North America | Greenland | 18.49 | 10.58 | -3.34 (-4.09, -2.58) | <0.001 |
| High-income North America | United States of America | 33.54 | 9.55 | -7.22 (-7.62, -6.81) | <0.001 |
| Southern Latin America | Argentina | 47.50 | 30.64 | -1.97 (-2.45, -1.49) | <0.001 |
| Southern Latin America | Chile | 47.40 | 26.40 | -2.48 (-2.76, -2.20) | <0.001 |
| Southern Latin America | Uruguay | 32.91 | 16.81 | -3.31 (-3.81, -2.81) | <0.001 |
| Western Europe | Andorra | 27.83 | 7.61 | -5.12 (-5.63, -4.61) | <0.001 |
| Western Europe | Austria | 45.56 | 13.72 | -5.75 (-5.94, -5.56) | <0.001 |
| Western Europe | Belgium | 45.18 | 12.69 | -6.22 (-6.63, -5.80) | <0.001 |
| Western Europe | Cyprus | 84.74 | 26.98 | -4.78 (-5.09, -4.46) | <0.001 |
| Western Europe | Denmark | 41.44 | 10.68 | -6.75 (-7.09, -6.41) | <0.001 |
| Western Europe | Finland | 15.40 | 2.15 | -9.06 (-9.68, -8.43) | <0.001 |
| Western Europe | France | 26.23 | 7.81 | -5.92 (-6.20, -5.63) | <0.001 |
| Western Europe | Germany | 43.03 | 12.67 | -5.70 (-6.11, -5.28) | <0.001 |
| Western Europe | Greece | 68.77 | 24.85 | -5.30 (-5.66, -4.94) | <0.001 |
| Western Europe | Iceland | 11.14 | 2.30 | -7.13 (-7.65, -6.61) | <0.001 |
| Western Europe | Ireland | 39.27 | 8.39 | -7.70 (-7.84, -7.57) | <0.001 |
| Western Europe | Israel | 53.71 | 19.26 | -4.81 (-4.95, -4.68) | <0.001 |
| Western Europe | Italy | 46.89 | 16.04 | -5.25 (-5.41, -5.09) | <0.001 |
| Western Europe | Luxembourg | 32.00 | 8.11 | -6.92 (-7.48, -6.35) | <0.001 |
| Western Europe | Malta | 48.84 | 14.55 | -5.55 (-6.05, -5.04) | <0.001 |
| Western Europe | Monaco | 34.73 | 12.66 | -4.85 (-5.22, -4.47) | <0.001 |
| Western Europe | Netherlands | 47.17 | 12.75 | -6.37 (-6.50, -6.23) | <0.001 |
| Western Europe | Norway | 21.35 | 3.45 | -8.29 (-8.56, -8.01) | <0.001 |
| Western Europe | Portugal | 39.85 | 8.81 | -7.87 (-8.13, -7.61) | <0.001 |
| Western Europe | San Marino | 18.59 | 6.48 | -3.42 (-4.43, -2.41) | <0.001 |
| Western Europe | Spain | 30.98 | 8.88 | -6.37 (-6.58, -6.16) | <0.001 |
| Western Europe | Sweden | 18.39 | 2.99 | -8.47 (-8.92, -8.01) | <0.001 |
| Western Europe | Switzerland | 30.20 | 6.95 | -6.65 (-7.01, -6.29) | <0.001 |
| Western Europe | United Kingdom | 44.60 | 11.53 | -6.76 (-7.09, -6.43) | <0.001 |
| Andean Latin America | Bolivia (Plurinational State of) | 85.67 | 48.36 | -2.72 (-3.26, -2.16) | <0.001 |
| Andean Latin America | Ecuador | 65.57 | 30.83 | -4.28 (-5.09, -3.46) | <0.001 |
| Andean Latin America | Peru | 59.97 | 37.24 | -2.18 (-2.85, -1.50) | <0.001 |
| Caribbean | Antigua and Barbuda | 54.71 | 43.71 | -0.62 (-0.86, -0.38) | <0.001 |
| Caribbean | Bahamas | 51.35 | 36.69 | -1.29 (-1.66, -0.91) | <0.001 |
| Caribbean | Barbados | 60.15 | 48.64 | -0.83 (-1.16, -0.49) | <0.001 |
| Caribbean | Belize | 75.74 | 49.15 | -1.58 (-2.12, -1.03) | <0.001 |
| Caribbean | Bermuda | 19.19 | 6.87 | -5.35 (-6.14, -4.55) | <0.001 |
| Caribbean | Cuba | 53.70 | 47.28 | -0.33 (-0.74, 0.09) | 0.118 |
| Caribbean | Dominica | 55.35 | 58.05 | 0.72 (0.34, 1.09) | 0.001 |
| Caribbean | Dominican Republic | 41.28 | 55.05 | 1.29 (0.97, 1.61) | <0.001 |
| Caribbean | Grenada | 78.98 | 71.11 | -0.88 (-1.13, -0.62) | <0.001 |
| Caribbean | Guyana | 82.71 | 87.72 | 0.20 (-0.01, 0.41) | 0.079 |
| Caribbean | Haiti | 25.44 | 24.40 | 0.02 (-0.26, 0.29) | 0.912 |
| Caribbean | Jamaica | 32.90 | 35.04 | 0.68 (0.10, 1.25) | 0.026 |
| Caribbean | Puerto Rico | 13.70 | 7.13 | -3.38 (-4.15, -2.60) | <0.001 |
| Caribbean | Saint Kitts and Nevis | 31.71 | 20.76 | -2.03 (-2.65, -1.39) | <0.001 |
| Caribbean | Saint Lucia | 66.02 | 48.92 | -1.72 (-2.35, -1.09) | <0.001 |
| Caribbean | Saint Vincent and the Grenadines | 73.47 | 63.97 | -0.38 (-0.72, -0.05) | 0.040 |
| Caribbean | Suriname | 83.46 | 68.74 | -0.60 (-1.15, -0.05) | 0.035 |
| Caribbean | Trinidad and Tobago | 91.55 | 65.14 | -1.60 (-2.05, -1.14) | <0.001 |
| Caribbean | United States Virgin Islands | 23.33 | 13.01 | -2.43 (-3.15, -1.71) | <0.001 |
| Central Latin America | Colombia | 50.29 | 22.60 | -3.72 (-3.87, -3.56) | <0.001 |
| Central Latin America | Costa Rica | 39.39 | 18.09 | -3.49 (-3.95, -3.02) | <0.001 |
| Central Latin America | El Salvador | 37.14 | 35.93 | 0.17 (-0.30, 0.64) | 0.447 |
| Central Latin America | Guatemala | 67.48 | 38.11 | -1.78 (-2.30, -1.26) | <0.001 |
| Central Latin America | Honduras | 39.18 | 41.51 | 0.72 (0.16, 1.29) | 0.022 |
| Central Latin America | Mexico | 66.28 | 36.73 | -2.50 (-2.93, -2.07) | <0.001 |
| Central Latin America | Nicaragua | 16.19 | 16.06 | -0.20 (-0.74, 0.34) | 0.504 |
| Central Latin America | Panama | 32.13 | 16.51 | -2.90 (-3.35, -2.45) | <0.001 |
| Central Latin America | Venezuela (Bolivarian Republic of) | 64.01 | 42.02 | -1.98 (-2.30, -1.65) | <0.001 |
| Tropical Latin America | Brazil | 39.41 | 22.47 | -2.88 (-3.15, -2.61) | <0.001 |
| Tropical Latin America | Paraguay | 22.03 | 21.98 | 0.63 (0.35, 0.91) | <0.001 |
| North Africa and Middle East | Afghanistan | 48.08 | 36.96 | 0.07 (-0.76, 0.92) | 0.785 |
| North Africa and Middle East | Algeria | 117.61 | 89.21 | -1.44 (-1.65, -1.24) | <0.001 |
| North Africa and Middle East | Bahrain | 293.99 | 147.96 | -4.16 (-4.64, -3.68) | <0.001 |
| North Africa and Middle East | Egypt | 240.53 | 236.50 | 0.12 (-0.47, 0.71) | 0.678 |
| North Africa and Middle East | Iran (Islamic Republic of) | 116.68 | 72.96 | -1.88 (-2.07, -1.69) | <0.001 |
| North Africa and Middle East | Iraq | 140.05 | 152.82 | -0.02 (-0.38, 0.35) | 0.967 |
| North Africa and Middle East | Jordan | 95.85 | 58.04 | -2.95 (-3.38, -2.52) | <0.001 |
| North Africa and Middle East | Kuwait | 113.54 | 76.29 | -2.73 (-3.30, -2.15) | <0.001 |
| North Africa and Middle East | Lebanon | 58.00 | 41.51 | -1.02 (-1.50, -0.53) | <0.001 |
| North Africa and Middle East | Libya | 91.89 | 94.61 | -0.05 (-0.65, 0.56) | 0.860 |
| North Africa and Middle East | Morocco | 91.94 | 91.52 | 0.22 (-0.20, 0.63) | 0.305 |
| North Africa and Middle East | Oman | 159.15 | 115.95 | -0.73 (-1.12, -0.34) | 0.002 |
| North Africa and Middle East | Palestine | 117.49 | 87.08 | -1.45 (-2.13, -0.76) | 0.001 |
| North Africa and Middle East | Qatar | 283.51 | 108.22 | -6.37 (-7.07, -5.67) | <0.001 |
| North Africa and Middle East | Saudi Arabia | 181.13 | 132.1 | -1.38 (-1.61, -1.15) | <0.001 |
| North Africa and Middle East | Sudan | 65.90 | 90.94 | 2.16 (1.88, 2.43) | <0.001 |
| North Africa and Middle East | Syrian Arab Republic | 129.66 | 121.82 | 0.22 (-0.16, 0.60) | 0.279 |
| North Africa and Middle East | Tunisia | 98.66 | 67.86 | -1.87 (-2.07, -1.68) | <0.001 |
| North Africa and Middle East | Turkey | 76.20 | 65.01 | 0.35 (-0.14, 0.84) | 0.184 |
| North Africa and Middle East | United Arab Emirates | 176.79 | 112.31 | -0.02 (-1.11, 1.09) | 0.834 |
| North Africa and Middle East | Yemen | 87.75 | 82.43 | 0.66 (0.30, 1.03) | 0.002 |
| South Asia | Bangladesh | 34.40 | 31.39 | 0.08 (-0.76, 0.94) | 0.793 |
| South Asia | Bhutan | 41.12 | 66.31 | 3.68 (2.90, 4.47) | <0.001 |
| South Asia | India | 49.70 | 82.07 | 3.89 (3.08, 4.72) | <0.001 |
| South Asia | Nepal | 47.88 | 54.64 | 2.26 (1.32, 3.20) | <0.001 |
| South Asia | Pakistan | 67.93 | 77.24 | 1.79 (1.06, 2.52) | <0.001 |
| East Asia | China | 94.15 | 99.09 | 0.55 (-0.05, 1.16) | 0.087 |
| East Asia | Democratic People's Republic of Korea | 45.36 | 29.88 | -1.66 (-2.20, -1.12) | <0.001 |
| East Asia | Taiwan (Province of China) | 51.66 | 22.40 | -3.27 (-3.83, -2.70) | <0.001 |
| Oceania | American Samoa | 24.41 | 23.49 | -0.67 (-1.19, -0.15) | 0.016 |
| Oceania | Cook Islands | 20.44 | 14.27 | -2.04 (-2.74, -1.33) | <0.001 |
| Oceania | Fiji | 68.27 | 70.81 | 0.44 (0.06, 0.83) | 0.032 |
| Oceania | Guam | 22.29 | 17.60 | 1.12 (-0.36, 2.62) | 0.120 |
| Oceania | Kiribati | 15.38 | 17.59 | 0.48 (0.11, 0.85) | 0.017 |
| Oceania | Marshall Islands | 40.89 | 39.10 | -0.69 (-0.98, -0.40) | <0.001 |
| Oceania | Micronesia (Federated States of) | 34.51 | 38.34 | 0.68 (0.54, 0.83) | <0.001 |
| Oceania | Nauru | 53.03 | 34.81 | -2.28 (-2.58, -1.98) | <0.001 |
| Oceania | Niue | 31.39 | 25.83 | -1.41 (-2.05, -0.77) | <0.001 |
| Oceania | Northern Mariana Islands | 32.23 | 28.31 | 0.78 (-0.28, 1.84) | 0.137 |
| Oceania | Palau | 29.73 | 29.49 | 1.85 (0.10, 3.64) | 0.029 |
| Oceania | Papua New Guinea | 33.09 | 35.65 | 0.60 (0.23, 0.97) | 0.004 |
| Oceania | Samoa | 29.45 | 38.6 | 1.24 (1.10, 1.39) | <0.001 |
| Oceania | Solomon Islands | 22.13 | 23.04 | 0.66 (0.28, 1.05) | 0.003 |
| Oceania | Tokelau | 22.81 | 19.96 | -1.68 (-2.43, -0.93) | <0.001 |
| Oceania | Tonga | 27.54 | 32.4 | 0.67 (0.36, 0.98) | <0.001 |
| Oceania | Tuvalu | 12.21 | 12.62 | -0.10 (-0.52, 0.33) | 0.699 |
| Oceania | Vanuatu | 15.36 | 25.94 | 4.01 (3.32, 4.72) | <0.001 |
| Southeast Asia | Cambodia | 28.35 | 38.47 | 0.97 (0.50, 1.44) | <0.001 |
| Southeast Asia | Indonesia | 52.80 | 64.56 | 1.37 (1.15, 1.60) | <0.001 |
| Southeast Asia | Lao People's Democratic Republic | 40.49 | 62.1 | 1.88 (1.33, 2.44) | <0.001 |
| Southeast Asia | Malaysia | 89.83 | 50.16 | -2.45 (-2.82, -2.08) | <0.001 |
| Southeast Asia | Maldives | 54.12 | 23.23 | -4.76 (-5.26, -4.25) | <0.001 |
| Southeast Asia | Mauritius | 32.72 | 22.87 | -2.78 (-3.52, -2.04) | <0.001 |
| Southeast Asia | Myanmar | 48.90 | 78.69 | 1.94 (1.46, 2.42) | <0.001 |
| Southeast Asia | Philippines | 59.49 | 57.11 | -0.48 (-0.91, -0.05) | 0.038 |
| Southeast Asia | Seychelles | 29.03 | 17.25 | -1.76 (-2.30, -1.21) | <0.001 |
| Southeast Asia | Sri Lanka | 41.92 | 46.13 | 0.78 (0.40, 1.15) | <0.001 |
| Southeast Asia | Thailand | 77.34 | 51.09 | -3.06 (-3.58, -2.54) | <0.001 |
| Southeast Asia | Timor-Leste | 26.75 | 44.73 | 3.68 (2.79, 4.58) | <0.001 |
| Southeast Asia | Viet Nam | 17.28 | 51.34 | 5.45 (4.96, 5.93) | <0.001 |
| Central Sub-Saharan Africa | Angola | 39.44 | 69.82 | 3.60 (3.21, 3.99) | <0.001 |
| Central Sub-Saharan Africa | Central African Republic | 35.56 | 28.47 | -0.49 (-0.80, -0.17) | 0.007 |
| Central Sub-Saharan Africa | Congo | 52.47 | 70.41 | 2.16 (1.81, 2.51) | <0.001 |
| Central Sub-Saharan Africa | Democratic Republic of the Congo | 19.81 | 19.10 | 0.03 (-0.20, 0.27) | 0.801 |
| Central Sub-Saharan Africa | Equatorial Guinea | 82.68 | 118.49 | 1.28 (0.89, 1.66) | <0.001 |
| Central Sub-Saharan Africa | Gabon | 119.10 | 107.11 | -0.55 (-0.77, -0.34) | <0.001 |
| Eastern Sub-Saharan Africa | Burundi | 23.73 | 15.18 | -1.92 (-2.28, -1.56) | <0.001 |
| Eastern Sub-Saharan Africa | Comoros | 12.52 | 10.12 | -1.33 (-1.62, -1.03) | <0.001 |
| Eastern Sub-Saharan Africa | Djibouti | 76.77 | 71.68 | 0.05 (-0.16, 0.27) | 0.579 |
| Eastern Sub-Saharan Africa | Eritrea | 48.14 | 30.99 | -1.56 (-1.80, -1.33) | <0.001 |
| Eastern Sub-Saharan Africa | Ethiopia | 17.59 | 14.95 | -0.33 (-0.57, -0.09) | 0.014 |
| Eastern Sub-Saharan Africa | Kenya | 15.44 | 22.00 | 2.02 (1.40, 2.63) | <0.001 |
| Eastern Sub-Saharan Africa | Madagascar | 10.76 | 11.55 | 0.93 (0.39, 1.47) | 0.004 |
| Eastern Sub-Saharan Africa | Malawi | 18.08 | 14.91 | -0.84 (-1.03, -0.65) | <0.001 |
| Eastern Sub-Saharan Africa | Mozambique | 13.29 | 15.42 | 1.43 (0.90, 1.95) | <0.001 |
| Eastern Sub-Saharan Africa | Rwanda | 26.76 | 13.71 | -2.86 (-3.26, -2.46) | <0.001 |
| Eastern Sub-Saharan Africa | Somalia | 20.86 | 15.75 | -0.72 (-1.21, -0.22) | 0.009 |
| Eastern Sub-Saharan Africa | South Sudan | 54.59 | 25.61 | -2.80 (-3.50, -2.10) | <0.001 |
| Eastern Sub-Saharan Africa | Uganda | 25.45 | 21.05 | -0.57 (-0.97, -0.18) | 0.010 |
| Eastern Sub-Saharan Africa | United Republic of Tanzania | 14.77 | 20.66 | 1.63 (1.31, 1.94) | <0.001 |
| Eastern Sub-Saharan Africa | Zambia | 25.60 | 35.05 | 2.70 (1.32, 4.10) | <0.001 |
| Southern Sub-Saharan Africa | Botswana | 77.82 | 62.53 | -0.70 (-1.43, 0.03) | 0.110 |
| Southern Sub-Saharan Africa | Eswatini | 82.85 | 74.08 | -0.14 (-0.53, 0.24) | 0.510 |
| Southern Sub-Saharan Africa | Lesotho | 28.71 | 44.21 | 2.45 (1.95, 2.96) | <0.001 |
| Southern Sub-Saharan Africa | Namibia | 62.79 | 73.57 | 1.12 (0.86, 1.39) | <0.001 |
| Southern Sub-Saharan Africa | South Africa | 73.29 | 68.64 | -0.10 (-0.60, 0.40) | 0.746 |
| Southern Sub-Saharan Africa | Zimbabwe | 32.56 | 24.33 | -1.51 (-1.77, -1.24) | <0.001 |
| Western Sub-Saharan Africa | Benin | 46.58 | 37.26 | 0.14 (-0.76, 1.04) | 0.759 |
| Western Sub-Saharan Africa | Burkina Faso | 37.94 | 35.69 | 0.11 (-0.43, 0.66) | 0.604 |
| Western Sub-Saharan Africa | Cabo Verde | 45.40 | 94.18 | 4.40 (3.03, 5.79) | <0.001 |
| Western Sub-Saharan Africa | Cameroon | 50.20 | 45.73 | 0.38 (-0.16, 0.92) | 0.171 |
| Western Sub-Saharan Africa | Chad | 43.94 | 42.06 | 0.63 (0.13, 1.13) | 0.023 |
| Western Sub-Saharan Africa | Côte d’Ivoire | 65.39 | 62.03 | 0.18 (-0.64, 1.01) | 0.649 |
| Western Sub-Saharan Africa | Gambia | 51.77 | 50.16 | 0.17 (-0.18, 0.52) | 0.331 |
| Western Sub-Saharan Africa | Ghana | 62.82 | 78.2 | 2.05 (1.28, 2.82) | <0.001 |
| Western Sub-Saharan Africa | Guinea | 42.15 | 39.08 | 0.30 (-0.16, 0.75) | 0.204 |
| Western Sub-Saharan Africa | Guinea-Bissau | 63.40 | 51.70 | -0.47 (-0.82, -0.13) | 0.015 |
| Western Sub-Saharan Africa | Liberia | 28.68 | 27.85 | 0.21 (-0.54, 0.97) | 0.532 |
| Western Sub-Saharan Africa | Mali | 39.43 | 37.34 | 0.50 (0.01, 1.00) | 0.059 |
| Western Sub-Saharan Africa | Mauritania | 78.92 | 81.31 | 0.63 (0.24, 1.01) | 0.004 |
| Western Sub-Saharan Africa | Niger | 48.80 | 38.66 | -1.04 (-1.56, -0.51) | 0.001 |
| Western Sub-Saharan Africa | Nigeria | 58.37 | 65.81 | 2.06 (1.12, 3.02) | <0.001 |
| Western Sub-Saharan Africa | Sao Tome and Principe | 24.23 | 49.46 | 3.52 (2.98, 4.06) | <0.001 |
| Western Sub-Saharan Africa | Senegal | 45.53 | 38.14 | -0.46 (-1.53, 0.63) | 0.306 |
| Western Sub-Saharan Africa | Sierra Leone | 34.65 | 32.49 | 0.06 (-0.56, 0.67) | 0.781 |
| Western Sub-Saharan Africa | Togo | 51.03 | 41.80 | 0.12 (-0.71, 0.96) | 0.814 |

Notes: * *P* value for trends test.

CI=confidence interval; EAPC=estimated annual percentage change.
