# Supplementary material for: Inequalities in greenspace access and their associations with all-cause and cause-specific mortality attributable to PM2.5: a global analysis from 2000 to 2020
Source: BMC Public Health. 2026 Jul 25;26:3293. doi: 10.1186/s12889-026-28654-w (PMC13749990; doi:10.1186/s12889-026-28654-w)
Supplement: Supplementary file 5 [file 12889_2026_28654_MOESM5_ESM.docx]

**Additional file 5*.*** Estimated annual percentage change (EAPC) in greenspace access, 2000-2020.

| **GBD regions** | **Countries and territories** | **Greenspace access (%)** | | **EAPC (%, 95% CI)** | ***P* value*** |
| --- | --- | --- | --- | --- | --- |
|  |  | **2000** | **2020** |  |  |
| Central Asia | Armenia | 32.3 | 25.0 | -1.23 (-1.29, -1.18) | <0.001 |
| Central Asia | Azerbaijan | 45.1 | 34.7 | -1.30 (-1.35, -1.25) | <0.001 |
| Central Asia | Georgia | 47.6 | 37.1 | -1.26 (-1.31, -1.21) | <0.001 |
| Central Asia | Kazakhstan | 63.2 | 52.2 | -0.75 (-0.85, -0.66) | <0.001 |
| Central Asia | Kyrgyzstan | 45.3 | 37.5 | -0.84 (-0.95, -0.73) | <0.001 |
| Central Asia | Mongolia | 82.9 | 57.3 | -1.96 (-2.30, -1.62) | <0.001 |
| Central Asia | Tajikistan | 37.6 | 32.5 | -0.55 (-0.73, -0.37) | <0.001 |
| Central Asia | Turkmenistan | 41.7 | 31.2 | -0.98 (-1.16, -0.79) | <0.001 |
| Central Asia | Uzbekistan | 27.2 | 18.0 | -1.18 (-1.50, -0.86) | <0.001 |
| Central Europe | Albania | 39.7 | 22.9 | -2.82 (-2.95, -2.70) | <0.001 |
| Central Europe | Bosnia and Herzegovina | 69.5 | 57.5 | -1.09 (-1.23, -0.95) | <0.001 |
| Central Europe | Bulgaria | 42.0 | 32.5 | -1.54 (-1.77, -1.31) | <0.001 |
| Central Europe | Croatia | 45.7 | 37.2 | -1.22 (-1.39, -1.06) | <0.001 |
| Central Europe | Czechia | 27.1 | 21.8 | -1.17 (-1.27, -1.07) | <0.001 |
| Central Europe | Hungary | 23.9 | 16.2 | -2.06 (-2.23, -1.90) | <0.001 |
| Central Europe | Montenegro | 68.4 | 58.5 | -0.88 (-0.98, -0.78) | <0.001 |
| Central Europe | North Macedonia | 56.5 | 52.5 | -0.56 (-0.79, -0.33) | <0.001 |
| Central Europe | Poland | 39.4 | 33.8 | -0.79 (-0.85, -0.72) | <0.001 |
| Central Europe | Romania | 34.4 | 19.7 | -2.33 (-2.83, -1.83) | <0.001 |
| Central Europe | Serbia | 34.5 | 29.7 | -0.86 (-0.96, -0.75) | <0.001 |
| Central Europe | Slovakia | 30.3 | 25.6 | -1.02 (-1.17, -0.88) | <0.001 |
| Central Europe | Slovenia | 37.9 | 33.2 | -0.57 (-0.70, -0.44) | <0.001 |
| Eastern Europe | Belarus | 33.0 | 21.3 | -2.18 (-2.35, -2.00) | <0.001 |
| Eastern Europe | Estonia | 27.9 | 19.5 | -1.57 (-1.83, -1.30) | <0.001 |
| Eastern Europe | Latvia | 45.3 | 37.0 | -1.02 (-1.10, -0.94) | <0.001 |
| Eastern Europe | Lithuania | 49.0 | 38.2 | -1.25 (-1.34, -1.17) | <0.001 |
| Eastern Europe | Republic of Moldova | 32.2 | 30.6 | -0.22 (-0.26, -0.19) | <0.001 |
| Eastern Europe | Russian Federation | 35.7 | 28.9 | -1.07 (-1.12, -1.01) | <0.001 |
| Eastern Europe | Ukraine | 28.4 | 24.1 | -0.94 (-1.05, -0.82) | <0.001 |
| Australasia | Australia | 14.2 | 9.9 | -1.72 (-1.95, -1.49) | <0.001 |
| Australasia | New Zealand | 22.7 | 18.5 | -1.05 (-1.17, -0.93) | <0.001 |
| High-income Asia Pacific | Brunei Darussalam | 47.5 | 38.2 | -1.20 (-1.26, -1.14) | <0.001 |
| High-income Asia Pacific | Japan | 20.8 | 10.1 | -4.73 (-5.36, -4.10) | <0.001 |
| High-income Asia Pacific | Republic of Korea | 26.2 | 16.0 | -3.18 (-3.47, -2.90) | <0.001 |
| High-income Asia Pacific | Singapore | 13.2 | 5.3 | -5.54 (-6.65, -4.42) | <0.001 |
| High-income North America | Canada | 15.1 | 10.6 | -1.70 (-1.83, -1.56) | <0.001 |
| High-income North America | Greenland | NA | NA | NA | NA |
| High-income North America | United States of America | 18.2 | 14.2 | -1.16 (-1.29, -1.03) | <0.001 |
| Southern Latin America | Argentina | 29.3 | 20.0 | -2.40 (-2.70, -2.10) | <0.001 |
| Southern Latin America | Chile | 37.0 | 25.4 | -2.29 (-2.54, -2.05) | <0.001 |
| Southern Latin America | Uruguay | 19.2 | 13.0 | -1.89 (-2.03, -1.74) | <0.001 |
| Western Europe | Andorra | 48.5 | 40.7 | -1.16 (-1.48, -0.84) | <0.001 |
| Western Europe | Austria | 27.3 | 21.5 | -1.19 (-1.30, -1.08) | <0.001 |
| Western Europe | Belgium | 16.8 | 9.5 | -2.85 (-3.09, -2.60) | <0.001 |
| Western Europe | Cyprus | 17.6 | 10.5 | -2.87 (-3.19, -2.55) | <0.001 |
| Western Europe | Denmark | 16.4 | 10.2 | -2.73 (-3.08, -2.38) | <0.001 |
| Western Europe | Finland | 35.3 | 27.3 | -1.83 (-2.08, -1.58) | <0.001 |
| Western Europe | France | 18.3 | 14.1 | -1.50 (-1.69, -1.30) | <0.001 |
| Western Europe | Germany | 22.2 | 14.5 | -2.26 (-2.45, -2.06) | <0.001 |
| Western Europe | Greece | 21.4 | 16.4 | -1.89 (-2.11, -1.67) | <0.001 |
| Western Europe | Iceland | 85.7 | 86.4 | -0.00 (-0.06, 0.05) | 0.924 |
| Western Europe | Ireland | 35.3 | 28.3 | -1.24 (-1.44, -1.04) | <0.001 |
| Western Europe | Israel | 19.6 | 14.4 | -1.35 (-1.80, -0.90) | <0.001 |
| Western Europe | Italy | 10.0 | 8.1 | -0.98 (-1.10, -0.85) | <0.001 |
| Western Europe | Luxembourg | 28.5 | 22.2 | -1.38 (-1.55, -1.22) | <0.001 |
| Western Europe | Malta | 13.1 | 9.9 | -1.36 (-1.41, -1.31) | <0.001 |
| Western Europe | Monaco | 5.0 | 3.6 | -2.06 (-2.33, -1.79) | <0.001 |
| Western Europe | Netherlands | 21.8 | 12.7 | -2.89 (-3.19, -2.60) | <0.001 |
| Western Europe | Norway | 49.3 | 35.8 | -1.91 (-2.14, -1.67) | <0.001 |
| Western Europe | Portugal | 18.3 | 13.9 | -1.07 (-1.26, -0.89) | <0.001 |
| Western Europe | San Marino | 16.6 | 15.1 | -0.45 (-0.49, -0.42) | <0.001 |
| Western Europe | Spain | 23.8 | 15.1 | -2.82 (-3.28, -2.35) | <0.001 |
| Western Europe | Sweden | 29.4 | 17.5 | -3.25 (-3.60, -2.89) | <0.001 |
| Western Europe | Switzerland | 22.4 | 16.7 | -1.56 (-1.75, -1.36) | <0.001 |
| Western Europe | United Kingdom | 11.9 | 6.9 | -2.87 (-3.39, -2.35) | <0.001 |
| Andean Latin America | Bolivia (Plurinational State of) | 70.8 | 53.4 | -1.92 (-2.12, -1.71) | <0.001 |
| Andean Latin America | Ecuador | 66.1 | 45.4 | -1.86 (-2.01, -1.72) | <0.001 |
| Andean Latin America | Peru | 51.7 | 34.0 | -2.13 (-2.25, -2.00) | <0.001 |
| Caribbean | Antigua and Barbuda | 30.5 | 22.2 | -1.53 (-1.76, -1.31) | <0.001 |
| Caribbean | Bahamas | 28.5 | 20.4 | -1.91 (-2.04, -1.77) | <0.001 |
| Caribbean | Barbados | 32.2 | 19.9 | -2.30 (-2.69, -1.91) | <0.001 |
| Caribbean | Belize | 80.9 | 72.5 | -0.63 (-0.72, -0.54) | <0.001 |
| Caribbean | Bermuda | 0.0 | 0.0 | NA | NA |
| Caribbean | Cuba | 55.2 | 52.3 | -0.32 (-0.42, -0.22) | <0.001 |
| Caribbean | Dominica | 73.2 | 61.0 | -1.00 (-1.15, -0.85) | <0.001 |
| Caribbean | Dominican Republic | 43.4 | 31.9 | -1.54 (-1.73, -1.36) | <0.001 |
| Caribbean | Grenada | 56.5 | 46.7 | -1.16 (-1.35, -0.97) | <0.001 |
| Caribbean | Guyana | 44.5 | 38.5 | -1.00 (-1.20, -0.80) | <0.001 |
| Caribbean | Haiti | 63.0 | 53.2 | -0.81 (-0.91, -0.72) | <0.001 |
| Caribbean | Jamaica | 52.4 | 44.1 | -1.03 (-1.11, -0.95) | <0.001 |
| Caribbean | Puerto Rico | 29.2 | 26.8 | -0.43 (-0.47, -0.38) | <0.001 |
| Caribbean | Saint Kitts and Nevis | 47.9 | 39.5 | -1.14 (-1.53, -0.75) | <0.001 |
| Caribbean | Saint Lucia | 56.2 | 47.6 | -0.91 (-1.00, -0.82) | <0.001 |
| Caribbean | Saint Vincent and the Grenadines | 93.0 | 43.9 | -2.97 (-3.58, -2.35) | <0.001 |
| Caribbean | Suriname | 54.9 | 48.3 | -0.70 (-0.76, -0.65) | <0.001 |
| Caribbean | Trinidad and Tobago | 53.0 | 44.0 | -0.98 (-1.05, -0.90) | <0.001 |
| Caribbean | United States Virgin Islands | 28.0 | 23.1 | -1.03 (-1.09, -0.97) | <0.001 |
| Central Latin America | Colombia | 64.5 | 49.3 | -1.69 (-1.92, -1.46) | <0.001 |
| Central Latin America | Costa Rica | 47.6 | 40.7 | -0.81 (-0.92, -0.71) | <0.001 |
| Central Latin America | El Salvador | 61.7 | 53.9 | -0.82 (-1.01, -0.64) | <0.001 |
| Central Latin America | Guatemala | 74.8 | 66.0 | -0.62 (-0.66, -0.58) | <0.001 |
| Central Latin America | Honduras | 75.1 | 63.0 | -1.00 (-1.11, -0.90) | <0.001 |
| Central Latin America | Mexico | 27.5 | 18.8 | -1.79 (-1.90, -1.69) | <0.001 |
| Central Latin America | Nicaragua | 75.3 | 69.4 | -0.50 (-0.59, -0.41) | <0.001 |
| Central Latin America | Panama | 57.9 | 43.2 | -1.61 (-1.75, -1.46) | <0.001 |
| Central Latin America | Venezuela (Bolivarian Republic of) | 66.5 | 54.3 | -1.14 (-1.22, -1.06) | <0.001 |
| Tropical Latin America | Brazil | 31.6 | 22.7 | -1.82 (-1.91, -1.73) | <0.001 |
| Tropical Latin America | Paraguay | 54.0 | 38.8 | -1.83 (-2.02, -1.64) | <0.001 |
| North Africa and Middle East | Afghanistan | 50.5 | 42.0 | -0.91 (-0.98, -0.84) | <0.001 |
| North Africa and Middle East | Algeria | 33.6 | 21.7 | -2.38 (-2.60, -2.15) | <0.001 |
| North Africa and Middle East | Bahrain | 4.0 | 0.8 | -6.81 (-7.36, -6.27) | <0.001 |
| North Africa and Middle East | Egypt | 16.2 | 7.4 | -3.65 (-4.45, -2.86) | <0.001 |
| North Africa and Middle East | Iran (Islamic Republic of) | 39.6 | 27.2 | -1.97 (-2.18, -1.76) | <0.001 |
| North Africa and Middle East | Iraq | 48.0 | 34.2 | -1.55 (-2.01, -1.08) | <0.001 |
| North Africa and Middle East | Jordan | 31.7 | 21.4 | -2.05 (-2.44, -1.66) | <0.001 |
| North Africa and Middle East | Kuwait | 1.5 | 1.3 | -0.79 (-1.01, -0.57) | <0.001 |
| North Africa and Middle East | Lebanon | 28.3 | 16.9 | -2.32 (-2.46, -2.19) | <0.001 |
| North Africa and Middle East | Libya | 21.9 | 16.2 | -1.52 (-1.81, -1.23) | <0.001 |
| North Africa and Middle East | Morocco | 33.4 | 23.9 | -1.73 (-1.85, -1.62) | <0.001 |
| North Africa and Middle East | Oman | 13.1 | 7.9 | -2.35 (-2.72, -1.97) | <0.001 |
| North Africa and Middle East | Palestine | 22.0 | 13.6 | -2.13 (-2.24, -2.02) | <0.001 |
| North Africa and Middle East | Qatar | 0.8 | 0.5 | -1.17 (-1.78, -0.55) | 0.002 |
| North Africa and Middle East | Saudi Arabia | 9.8 | 7.9 | -0.92 (-1.00, -0.84) | <0.001 |
| North Africa and Middle East | Sudan | 51.4 | 43.9 | -0.81 (-0.86, -0.76) | <0.001 |
| North Africa and Middle East | Syrian Arab Republic | 33.4 | 26.9 | -1.05 (-1.16, -0.94) | <0.001 |
| North Africa and Middle East | Tunisia | 31.7 | 22.8 | -1.59 (-1.77, -1.42) | <0.001 |
| North Africa and Middle East | Turkey | 36.9 | 18.9 | -3.40 (-3.89, -2.91) | <0.001 |
| North Africa and Middle East | United Arab Emirates | 3.7 | 2.6 | -1.71 (-2.57, -0.85) | 0.002 |
| North Africa and Middle East | Yemen | 60.5 | 55.3 | -0.48 (-0.54, -0.41) | <0.001 |
| South Asia | Bangladesh | 26.2 | 21.7 | -0.84 (-0.90, -0.77) | <0.001 |
| South Asia | Bhutan | 91.6 | 84.0 | -0.41 (-0.45, -0.37) | <0.001 |
| South Asia | India | 33.3 | 23.9 | -1.58 (-1.67, -1.49) | <0.001 |
| South Asia | Nepal | 58.2 | 29.6 | -3.17 (-3.39, -2.96) | <0.001 |
| South Asia | Pakistan | 28.6 | 20.1 | -1.67 (-1.83, -1.51) | <0.001 |
| East Asia | China | 38.6 | 25.2 | -2.29 (-2.40, -2.18) | <0.001 |
| East Asia | Democratic People's Republic of Korea | 56.6 | 46.8 | -0.89 (-0.94, -0.84) | <0.001 |
| East Asia | Taiwan (Province of China) | 14.2 | 9.3 | -2.61 (-2.80, -2.43) | <0.001 |
| Oceania | American Samoa | 0.0 | 0.0 | NA | NA |
| Oceania | Cook Islands | 0.0 | 0.0 | NA | NA |
| Oceania | Fiji | 47.2 | 42.7 | -0.39 (-0.43, -0.36) | <0.001 |
| Oceania | Guam | 0.0 | 0.0 | NA | NA |
| Oceania | Kiribati | 0.0 | 0.0 | NA | NA |
| Oceania | Marshall Islands | 0.0 | 0.0 | NA | NA |
| Oceania | Micronesia (Federated States of) | 0.0 | 0.0 | NA | NA |
| Oceania | Nauru | 0.0 | 0.0 | NA | NA |
| Oceania | Niue | 0.0 | 0.0 | NA | NA |
| Oceania | Northern Mariana Islands | 0.0 | 0.0 | NA | NA |
| Oceania | Palau | 57.1 | 58.8 | 0.04 (-0.06, 0.14) | 0.413 |
| Oceania | Papua New Guinea | 94.5 | 92.9 | -0.08 (-0.09, -0.06) | <0.001 |
| Oceania | Samoa | 0.0 | 0.0 | NA | NA |
| Oceania | Solomon Islands | 67.2 | 63.2 | -0.33 (-0.41, -0.25) | <0.001 |
| Oceania | Tokelau | 0.0 | 0.0 | NA | NA |
| Oceania | Tonga | 0.0 | 0.0 | NA | NA |
| Oceania | Tuvalu | 0.0 | 0.0 | NA | NA |
| Oceania | Vanuatu | 74.6 | 63.2 | -0.76 (-0.83, -0.70) | <0.001 |
| Southeast Asia | Cambodia | 22.1 | 26.6 | 0.97 (0.88, 1.06) | <0.001 |
| Southeast Asia | Indonesia | 48.1 | 39.8 | -0.94 (-1.07, -0.82) | <0.001 |
| Southeast Asia | Lao People's Democratic Republic | 63.2 | 56.6 | -0.51 (-0.53, -0.48) | <0.001 |
| Southeast Asia | Malaysia | 46.9 | 32.5 | -1.94 (-2.02, -1.86) | <0.001 |
| Southeast Asia | Maldives | 3.8 | 5.0 | 1.69 (0.77, 2.63) | <0.001 |
| Southeast Asia | Mauritius | 0.0 | 0.0 | NA | NA |
| Southeast Asia | Myanmar | 37.1 | 33.6 | -0.51 (-0.59, -0.43) | <0.001 |
| Southeast Asia | Philippines | 43 | 35.1 | -0.96 (-1.00, -0.92) | <0.001 |
| Southeast Asia | Seychelles | 0.6 | 0.4 | -1.69 (-2.20, -1.17) | <0.001 |
| Southeast Asia | Sri Lanka | 59.4 | 44.9 | -1.25 (-1.35, -1.15) | <0.001 |
| Southeast Asia | Thailand | 31.4 | 23.8 | -1.41 (-1.52, -1.30) | <0.001 |
| Southeast Asia | Timor-Leste | 76.2 | 57.7 | -1.40 (-1.51, -1.29) | <0.001 |
| Southeast Asia | Viet Nam | 33.4 | 26.4 | -1.14 (-1.22, -1.06) | <0.001 |
| Central Sub-Saharan Africa | Angola | 92.9 | 47.6 | -3.32 (-3.65, -2.99) | <0.001 |
| Central Sub-Saharan Africa | Central African Republic | 80.3 | 74.8 | -0.34 (-0.38, -0.31) | <0.001 |
| Central Sub-Saharan Africa | Congo | 68.8 | 70.7 | 0.22 (-0.05, 0.49) | 0.119 |
| Central Sub-Saharan Africa | Democratic Republic of the Congo | 81.0 | 72.8 | -0.43 (-0.49, -0.38) | <0.001 |
| Central Sub-Saharan Africa | Equatorial Guinea | 95.4 | 88.6 | -0.29 (-0.34, -0.24) | <0.001 |
| Central Sub-Saharan Africa | Gabon | 76.4 | 74.8 | -0.11 (-0.12, -0.09) | <0.001 |
| Eastern Sub-Saharan Africa | Burundi | 75.7 | 64.8 | -0.75 (-0.81, -0.68) | <0.001 |
| Eastern Sub-Saharan Africa | Comoros | 88.4 | 81.3 | -0.49 (-0.53, -0.45) | <0.001 |
| Eastern Sub-Saharan Africa | Djibouti | 16.4 | 16.1 | -0.26 (-0.38, -0.14) | <0.001 |
| Eastern Sub-Saharan Africa | Eritrea | 65.0 | 64.5 | -0.09 (-0.11, -0.07) | <0.001 |
| Eastern Sub-Saharan Africa | Ethiopia | 58.7 | 53.8 | -0.46 (-0.51, -0.42) | <0.001 |
| Eastern Sub-Saharan Africa | Kenya | 45.9 | 40.0 | -0.68 (-0.77, -0.59) | <0.001 |
| Eastern Sub-Saharan Africa | Madagascar | 85.6 | 77.2 | -0.49 (-0.51, -0.47) | <0.001 |
| Eastern Sub-Saharan Africa | Malawi | 55.4 | 43.2 | -1.17 (-1.25, -1.10) | <0.001 |
| Eastern Sub-Saharan Africa | Mozambique | 77.9 | 65.6 | -0.87 (-0.92, -0.83) | <0.001 |
| Eastern Sub-Saharan Africa | Rwanda | 47.1 | 38.9 | -0.95 (-1.05, -0.85) | <0.001 |
| Eastern Sub-Saharan Africa | Somalia | 76.3 | 71.9 | -0.26 (-0.27, -0.24) | <0.001 |
| Eastern Sub-Saharan Africa | South Sudan | 99.0 | 97.9 | -0.07 (-0.07, -0.06) | <0.001 |
| Eastern Sub-Saharan Africa | Uganda | 58.4 | 44.8 | -1.30 (-1.33, -1.27) | <0.001 |
| Eastern Sub-Saharan Africa | United Republic of Tanzania | 60.8 | 45.0 | -1.48 (-1.55, -1.40) | <0.001 |
| Eastern Sub-Saharan Africa | Zambia | 74.1 | 59.3 | -1.20 (-1.26, -1.14) | <0.001 |
| Southern Sub-Saharan Africa | Botswana | 80.3 | 70.3 | -0.74 (-0.80, -0.68) | <0.001 |
| Southern Sub-Saharan Africa | Eswatini | 80.6 | 72.5 | -0.49 (-0.53, -0.44) | <0.001 |
| Southern Sub-Saharan Africa | Lesotho | 70.8 | 63.8 | -0.51 (-0.55, -0.47) | <0.001 |
| Southern Sub-Saharan Africa | Namibia | 62.7 | 45.5 | -1.60 (-1.62, -1.58) | <0.001 |
| Southern Sub-Saharan Africa | South Africa | 40.6 | 23.2 | -2.67 (-2.80, -2.54) | <0.001 |
| Southern Sub-Saharan Africa | Zimbabwe | 67.0 | 58.1 | -0.77 (-0.87, -0.67) | <0.001 |
| Western Sub-Saharan Africa | Benin | 57.8 | 52.0 | -0.51 (-0.55, -0.47) | <0.001 |
| Western Sub-Saharan Africa | Burkina Faso | 81.8 | 64.1 | -1.24 (-1.36, -1.12) | <0.001 |
| Western Sub-Saharan Africa | Cabo Verde | 87.7 | 87.8 | -0.00 (-0.04, 0.03) | 0.995 |
| Western Sub-Saharan Africa | Cameroon | 75.4 | 57.0 | -1.42 (-1.56, -1.27) | <0.001 |
| Western Sub-Saharan Africa | Chad | 76.6 | 64.4 | -0.89 (-0.94, -0.84) | <0.001 |
| Western Sub-Saharan Africa | Côte d’Ivoire | 72.8 | 60.2 | -0.95 (-1.01, -0.88) | <0.001 |
| Western Sub-Saharan Africa | Gambia | 52 | 35.4 | -1.72 (-1.88, -1.55) | <0.001 |
| Western Sub-Saharan Africa | Ghana | 63.2 | 47.7 | -1.37 (-1.50, -1.25) | <0.001 |
| Western Sub-Saharan Africa | Guinea | 81.0 | 72.0 | -0.53 (-0.58, -0.48) | <0.001 |
| Western Sub-Saharan Africa | Guinea-Bissau | 80.9 | 67.6 | -0.84 (-0.88, -0.80) | <0.001 |
| Western Sub-Saharan Africa | Liberia | 80.8 | 67.5 | -1.01 (-1.06, -0.96) | <0.001 |
| Western Sub-Saharan Africa | Mali | 64.4 | 49.8 | -1.23 (-1.26, -1.19) | <0.001 |
| Western Sub-Saharan Africa | Mauritania | 56.1 | 48.7 | -0.66 (-0.76, -0.55) | <0.001 |
| Western Sub-Saharan Africa | Niger | 51.6 | 43.4 | -0.80 (-0.86, -0.73) | <0.001 |
| Western Sub-Saharan Africa | Nigeria | 52.5 | 42.2 | -1.02 (-1.08, -0.96) | <0.001 |
| Western Sub-Saharan Africa | Sao Tome and Principe | 0.0 | 0.0 | NA | NA |
| Western Sub-Saharan Africa | Senegal | 49.2 | 40.7 | -0.94 (-0.96, -0.92) | <0.001 |
| Western Sub-Saharan Africa | Sierra Leone | 80.8 | 66.3 | -1.05 (-1.10, -1.01) | <0.001 |
| Western Sub-Saharan Africa | Togo | 58.0 | 44.4 | -1.29 (-1.40, -1.19) | <0.001 |

Notes: * *P* value for trends test. NA indicated that the estimated greenspace access in the country and territory was close to 0, and the EAPC and *P* values for trend could not be calculated. CI=confidence interval.
