# Supplementary material for: Inequalities in greenspace access and their associations with all-cause and cause-specific mortality attributable to PM2.5: a global analysis from 2000 to 2020
Source: BMC Public Health. 2026 Jul 25;26:3293. doi: 10.1186/s12889-026-28654-w (PMC13749990; doi:10.1186/s12889-026-28654-w)
Supplement: Supplementary file 6 [file 12889_2026_28654_MOESM6_ESM.docx]

**Additional file 6*.*** Interannual variation in the Gini coefficient of greenspace access, 2000-2020.

| **Location** | **Gini coefficient** | | | | | **EAPC (95% CI)** |
| --- | --- | --- | --- | --- | --- | --- |
|  | **2000** | **2005** | **2010** | **2015** | **2000** |  |
| SDI |  |  |  |  |  |  |
| High SDI | 0.173 | 0.182 | 0.189 | 0.192 | 0.189 | **0.503 (0.389, 0.617)** |
| Low SDI | 0.197 | 0.210 | 0.216 | 0.230 | 0.244 | **0.994 (0.929, 1.060)** |
| GBD super regions | | | | | |  |
| Central Europe, Eastern Europe,  and Central Asia | 0.117 | 0.125 | 0.128 | 0.132 | 0.149 | **0.891 (0.759, 1.023)** |
| High-income | 0.131 | 0.142 | 0.153 | 0.135 | 0.136 | -0.150 (-0.538, 0.239) |
| Latin America and Caribbean | 0.202 | 0.217 | 0.228 | 0.233 | 0.244 | **0.859 (0.782, 0.935)** |
| North Africa and Middle East | 0.210 | 0.230 | 0.243 | 0.283 | 0.323 | **2.143 (1.929, 2.359)** |
| South Asia | 0.044 | 0.040 | 0.035 | 0.032 | 0.030 | **-1.936 (-2.057, -1.815)** |
| Southeast Asia, East Asia, and Oceania | 0.066 | 0.067 | 0.083 | 0.094 | 0.109 | **2.934 (2.727, 3.142)** |
| Sub-Saharan Africa | 0.128 | 0.139 | 0.143 | 0.147 | 0.152 | **0.739 (0.663, 0.815)** |

Note: Bold within the table means the estimated annual percent change (EAPC) is statistically significant. CI=confidence interval. SDI=Socio-demographic Index.
