# Supplementary material for: Inequalities in greenspace access and their associations with all-cause and cause-specific mortality attributable to PM2.5: a global analysis from 2000 to 2020
Source: BMC Public Health. 2026 Jul 25;26:3293. doi: 10.1186/s12889-026-28654-w (PMC13749990; doi:10.1186/s12889-026-28654-w)
Supplement: Supplementary file 7 [file 12889_2026_28654_MOESM7_ESM.docx]

**Additional file 7*.*** Sensitivity analysis for the association between greenspace access and all cause and cause specific mortality attributable to ambient PM_2.5_.

Note: Statistically significant risk factors are in red, and two-sided *P* values < 0.05 were considered statistically significant.
